# ﻿Molecular and morphological data support the synonymy of *Muricanthusradix* Gmelin, 1791 and *Muricanthusambiguus* Reeve, 1845 (Gastropoda, Muricidae)

**DOI:** 10.3897/zookeys.1239.143837

**Published:** 2025-05-28

**Authors:** Francisco Morinha, James Ernest, Ana Archer-Taveira, Ana M. Rocha, Robert T. Iwamasa

**Affiliations:** 1 Morinha Lab- Laboratory of Biodiversity and Molecular Genetics, 5000-051 Vila Real, Portugal Morinha Lab- Laboratory of Biodiversity and Molecular Genetics Vila Real Portugal; 2 P.O. Box 0833-0034, Panama City, Panama Unaffiliated Panama City Panama; 3 i3S—Instituto de Investigação e Inovação em Saúde, Universidade do Porto, 4200-135 Porto, Portugal Universidade do Porto Porto Portugal; 4 5201 Campau Drive, Midland, Michigan, USA Unaffiliated Midland United States of America

**Keywords:** ddRADseq, mithocondrial genes, morphometrics, *Muricanthus*, nuclear genes, phylogeny, taxonomy

## Abstract

The *Muricanthusradix*/*ambiguus*/*nigritus* complex includes species with a great diversity of shell shapes and shared habitats in various regions, which has raised questions and doubts about the current taxonomic classification of these species. *Muricanthusnigritus*, *M.radix*, and *M.ambiguus* are three similar-looking black and white murex found commonly on the west coast of North and South America. The wide variety of morphological patterns within and between these species makes the classification of specimens difficult by visual observation. To this day, controversy persists over whether *M.radix* and *M.ambiguus* are one or two distinct species. Molecular genetic data have helped clarify the taxonomic classification of many mollusk species in recent decades, contributing to a more accurate understanding of biodiversity and ecosystems. In this study, DNA barcoding and double digest restriction-site associated DNA sequencing (ddRAD-seq) methodologies were applied to complement morphological data, establishing for the first time the phylogenetic relationships between *M.nigritus*, *M.ambiguus* and *M.radix*. The classic mitochondrial and nuclear barcodes obtained from 80 specimens collected from three different geographic locations differentiated only two phylogenetic clades (*M.nigritus* and *M.radix/ambiguus* from Mexico differentiated from *M.radix*/*ambiguus* from Mexico and Panama). High levels of mitochondrial DNA introgression have been observed between *M.nigritus* and *M.radix/ambiguus*. The deep-level approach performed using 3692 loci obtained from ddRAD-seq also differentiated only two genetic clusters (*M.nigritus* and *M.radix/ambiguus*). Our results clearly support the proposal that *M.ambiguus* should be synonymized with *M.radix*.

## ﻿Introduction

The Muricidae (Gastropoda, Muricoidea) is a family that includes nearly 1800 extant species distributed throughout the world, exhibiting a very high and complex taxonomic diversity (Barco et al. 2010; Russini et al. 2023). The genus *Muricanthus* Swainson, 1840 comprises five species proposed according to a morphology-based taxonomy (*M.ambiguus* Reeve, 1845; *M.callidinus* Berry, 1958; *M.nigritus* Philippi, 1845; *M.radix* Gmelin, 1791; and *M.strausi* Verrill, 1950) (Houart and Wiedrick 2021). The assignment of species names for different-looking, but similar, black and white murex has been the subject of confusion for many years due to considerable variations seen in the shell structure (Pittman 2023). The difficulty and confusion over the proper identification of the shells, whose binomial names were established as *M.nigritus*, *M.radix*, and *M.ambiguus* have led to the misidentification of many shells in scientific publications and in those discussed or sold on the internet (GBIF Secretariat 2023a, 2023b, 2023c; Iwamasa 2023; Pittman 2023).

*Muricanthusnigritus* is commonly found in the northern region of Baja California in the Gulf of California and as far west as San Carlos, Baja Sur, Mexico (Houart and Wiedrick 2021; Iwamasa 2023). This species is best known as a delicacy in the Asian market and has been extensively written about due to over harvesting (Cudney-Bueno and Rowell 2008). *Muricanthusnigritus* is characterized by a pyriform shape, a heavy shell; a high spire; a long siphonal canal; horn-shaped spines with many spiral cords and its four midsection spiral cords are small; a high number of varices, generally 6–12; and with a small or missing labral tooth (Houart and Wiedrick 2021; Iwamasa 2023).

*Muricanthusradix* is typically found from as far south as Peru to Panama and Costa Rica. Some specimens have been documented as far north as Manzanillo in the Sea of Cortez, on the west coast of central Mexico (Houart and Wiedrick 2021). *Muricanthusradix* is characterized by a globose, round, and heavy shell; short spire; short siphonal canal; numerous spiral cords with numerous open flowery spines; a high number of varices, generally 8–14; and has a labral tooth (Houart and Wiedrick 2021).

*Muricanthusambiguus* has been found along the southern west coast of Mexico, extending further south to Panama. *Muricanthusambiguus* is characterized by a pyriform-shaped shell; it weighs less than *M.radix* of the same size; has a higher spire; a longer siphonal canal; numerous spiral cords and numerous, larger, open, flowery spines (by comparison with the two former species); shell sometimes sharper, with fewer varices (by comparison with the two previous species), generally 6–10; and a labral tooth (Houart and Wiedrick 2021). Both *M.radix* and *M.ambiguus* are known to cohabitate in colonies (Houart and Wiedrick 2021; Pittman 2023). Considerable variations in shell structure occur within the species, making it difficult to identify based solely on morphology-based taxonomic descriptions (Pittman 2023). Since the early days of their descriptions to the present, there has been controversy over whether *M.radix* and *M.ambiguus* are one species or two (Pittman 2023).

Marine gastropods shell shape and size can be affected by genetic factors and several anthropogenic factors (e.g., chemical contamination and climatic changes) and non-anthropogenic factors (e.g., food availability, predatory pressure, salinity, temperature, hydrodynamics, desiccation, and substrate type) (e.g., Zimmerman and Pechenik 1991; Verhaegen et al. 2018; Harayashiki et al. 2020a, 2020b). The diversity and complexity of shell morphology of many gastropod species have made taxonomic classification difficult using morphometric data alone (e.g., Pfenninger et al. 2006; Kantor et al. 2017, 2022; Wu et al. 2022). This can lead to an overestimation or underestimation of the number of species, depending on local environmental conditions the regions from which the specimens are characterized (Whelan 2021). Correct species assignment is of paramount importance in various fields of biological research, including, but not limited to, biodiversity, evolution, conservation, and phylogeographic studies (De Carvalho et al. 2008). In this context, the application of molecular genetic approaches has proven to be very useful in complementing and/or overcoming the morphology-based taxonomy limitations in different organisms, including various mollusk species (Xu et al. 2024). DNA barcoding methodologies have been widely applied as a complement to traditional taxonomy in the classification of several gastropods (e.g., Borges et al. 2016; Ran et al. 2020). Nevertheless, the historical and/or recent hybridization events, along with the absence of a barcoding gap among closely-related species, may introduce bias in taxonomic classifications using DNA barcoding technology (Phillips et al. 2022). Advances in high-throughput Next Generation Sequencing (NGS) technologies have enabled the development of several cost-effective strategies for rapid genotyping of thousands of markers from the whole genome of any organism (da Fonseca et al. 2016). The application of these methodologies in biodiversity and conservation studies has increased significantly in recent years (Theissinger et al. 2023). Double digest restriction-site associated DNA sequencing (ddRAD-seq) is an NGS-based approach that allows for the discovery and genotyping of thousands of SNPs, even without the organism’s reference genome (Peterson et al. 2012). It is widely used in biological studies, including taxonomic classifications of a broad range of taxa (e.g., Peterson et al. 2012; Andrews et al. 2016; Ivanov et al. 2021; Paran et al. 2023). The development of integrative taxonomic approaches is crucial for enhancing and clarifying our understanding of species complexes (Pante et al. 2015; Karbstein et al. 2024).

In this study, we applied molecular techniques (DNA barcoding and ddRADseq) to clarify the discrepancies, confusion, and controversies associated with the taxonomic classification of three *Muricanthus* species. We obtained morphological and genetic data on *M.nigritus*, *M.radix*, and *M.ambiguus* specimens collected from different geographic areas for the first time. The implications of the new molecular findings for species assignment are discussed in light of the morphometric data of the specimens and classic taxonomic descriptions. The relationship between *M.radix* and *M.ambiguus* is clarified, highlighting the importance of developing future studies on species boundaries and population genetics.

## ﻿Material and methods

### ﻿Specimen collection

Samples included in this study were obtained opportunistically, through the collaboration with various fishing vessels between November 2021 and March 2023. A total of 80 *Muricanthus* specimens were sampled in different regions (Isla Cebaco, Panama; Jalisco, Mexico; and Magdalena Bay, Baja Sur, Mexico; Fig, 1) and collection sites (see approximate coordinates in Suppl. material 1). Photographs of six positions (dorsal, ventral, anterior, posterior, left and right sides) for each specimen were made using an iPhone 15 cell phone camera. Measurements on length, weight, and number of varices were recorded for all specimens. Weight measurements were made on cleaned shells, where encrustations were removed as best as possible using a sonic cleaner (VRN Ultrasonic Scaler, Model number VRN-A8) and dental tools. Cleaning time varied depending on the amount of encrustation, generally ranging from 25 minutes to 1 hour. The length measurements were made using a digital caliper from the apex to the tail end. Classification based on previous taxonomic descriptions (Houart and Wiedrick 2021) was carried out where possible. In addition, four samples of *Hexaplexprinceps* Broderip, 1833, and five samples of *Phyllonotusregius* Swainson, 1821, used as outgroups in the phylogenetic tree were collected in Panama (see approximate coordinates in Suppl. material 1). Approximately 500 mg of foot tissue was collected from the specimens, placed in glass vials containing absolute ethanol and stored at -20 °C in order to preserve the samples for molecular DNA analysis.

**Figure 1.**
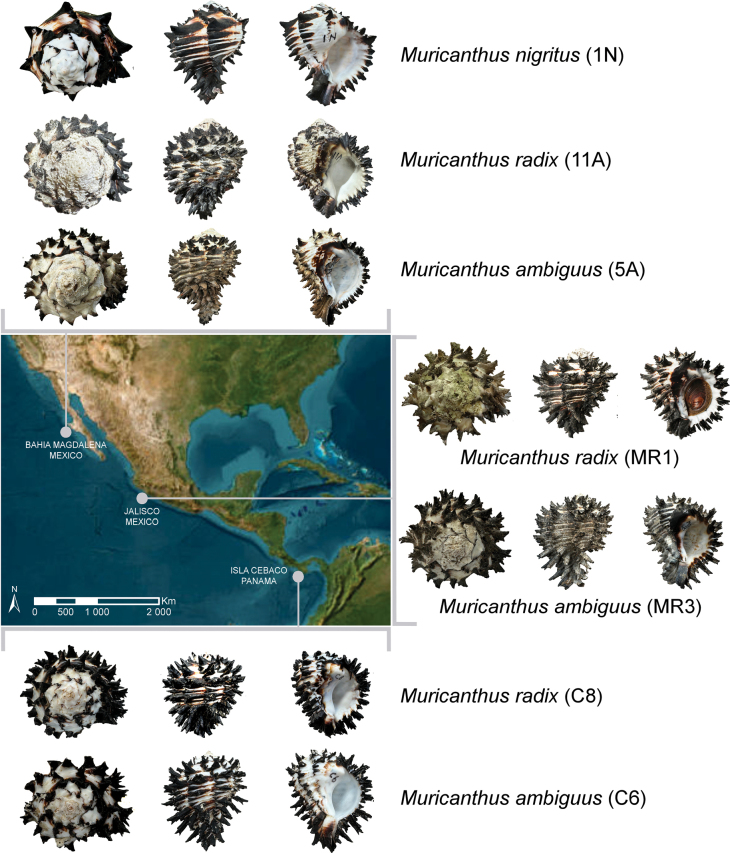
Geographic representation of sampling locations with some of the specimens collected at these sites (the dimensions are not to scale). Map was generated with ArcGIS 10.5 software (ESRI, Redland, USA, https://desktop.arcgis.com/en/).

### ﻿DNA extraction, amplification, and Sanger sequencing

DNA was isolated from foot tissues using the *Quick*-DNA Miniprep Plus Kit (Zymo Research, Irvine, CA, USA) following the manufacturer’s protocols with some optimizations. The digestion mixture contained 180 µL of water, 180 µL of solid tissue buffer, 40 µL of proteinase K and 300 mg of tissue shredded with a scalpel. The incubation step was performed at 55 °C for 24 h. The DNA was eluted in 60 µL of elution buffer. DNA concentration and integrity were evaluated with the fluorimeter Qubit 3.0 using the Invitrogen Qubit dsDNA High-Sensitivity (HS) assay kit (Thermo Fisher Scientific, USA) and agarose gels of genomic DNA.

Three different regions of the mitochondrial DNA (cytochrome oxidase I – *COI*, 12S ribosomal RNA (rRNA) and *16S rRNA* genes) and one fragment of the *28S rDNA* nuclear gene were PCR-amplified with primers previously characterized (Table 1). Each reaction included 10 μL of 2× MyTaq HS Mix (Meridian Bioscience, USA), 2.5 µM of each primer, 20 ng of template DNA, and ultrapure DNase/RNase-free water to make a total volume of 20 μl. The PCR run consisted of 95 °C for 5 min followed by 40 cycles of 95 °C for 30 s, annealing at variable temperatures depending on the primers (Table 1) for 1 min, 72 °C for 30 s, and a final extension at 60 °C for 10 min. All samples were bi-directionally sequenced by Sanger sequencing. Sequences were visualized and edited using the Chromas software version 2.6.6 (https://technelysium.com.au/wp/chromas/). All sequences were submitted to GenBank (see accession numbers in Suppl. material 1).

**Table 1.** Characterization of the primers used to amplify the four gene regions analyzed.

Gene region	Primer sequences (5′–3′)	T_a_ (°C)	Amplicon length (bp)	Reference
*COI*	F: CWAATCAYAAAGATATTGGAAC	50	~ 660	Colgan et al. 2003
	R: AATATAWACTTCWGGGTGACC			
*12S rRNA*	F: TGCCAGCAGYCGCGGTTA	58	~ 560	Oliverio and Mariottini 2001; Bandyopadhyay et al. 2008
	R: AGAGYGRCGGGCGATGTGT			
*16S rRNA*	F: CGCCTGTTTATCAAAAACAT	60	~ 530	Palumbi et al. 2001
	R: CCGGTCTGAACTCAGATCACGT			
*28S rDNA*	F: TAGGTCGACCCGCTGAAYTTAAGCA	58	~ 1430	Littlewood et al. 2000; Williams et al. 2003
	R: AGCGCCATCCATTTTCAGG			

F – forward; R – Reverse; T_a_ – annealing temperature

### ﻿Phylogenetic analyses

Median-joining networks were constructed for *COI*, *12S rRNA*, *16S rRNA*, and *28S rDNA* haplotypes using the program NETWORK v. 5.0.1.1 (Bandelt et al. 1999). The program BEAST v. 2.6.0 (Bouckaert et al. 2014) was used to construct a Bayesian maximum clade credibility (MCC) tree using individual and concatenated sequences of the four gene regions characterized for the specimens analyzed. The software JMODELTEST v. 2.1.7 (Darriba et al. 2012) was applied to estimate the best model of sequence evolution for each gene region (Table 2). The proportion of invariable sites and the alpha shape parameter (α) used to construct the phylogenetic tree are present in Table 2. The strict clock and Yule model priors were applied, with a normal distribution for the rate prior, letting the program estimate the mutation rates. The software run consisted of 100 million steps, with a sampling of the chains every 1,000 steps and a burn-in of 10%. The proper convergence and mixing of the chains, and effective sample sizes (ESS) were evaluated with TRACER v. 1.7.1 (Rambaut et al. 2018). The consensus tree was visuali­zed and edited with FIGTREE v. 1.4.4 (Rambaut 2018).

**Table 2.** Evolutionary models used in phylogenetic inference.

Gene region	Best fit model	α^a^	I^b^	Reference
*COI*	HKY+G	0.108	–	Hasegawa et al. 1985
*12S rRNA*	HKY+I	–	0.776	Hasegawa et al. 1985
*16S rRNA*	HKY+I	–	0.841	Hasegawa et al. 1985
*28S rDNA*	HKY+I	–	0.951	Hasegawa et al. 1985

^a^ alpha shape parameter; ^b^ proportion of invariable sites

### ﻿Species delimitation

The DNA-based species delimitation analyses were conducted using the ASAP (Assemble Species by Automatic Partitioning) distance-based method (Puillandre et al. 2021). The *COI* datasets were used for the ASAP analyses through its web server (https://bioinfo.mnhn.fr/abi/public/asap/, accessed on 22 March 2025), applying the default settings and the Kimura two-parameter (K2P) substitution model. The first analysis included the complete *COI* dataset with the 89 sequences including the outgroup species (*M.nigritus*, *M.radix*, *M.ambiguus*, *H.princeps*, and *P.regius*). The second analysis included only the *COI* sequences obtained from the 80 *Muricanthus* specimens.

### ﻿ddRADseq library preparation, sequencing, and data analysis

The ddRADseq analysis included only those specimens from which it was possible to obtain sufficient amounts of DNA and that passed the sample quality controls recommended for the protocol. Thus, a representative sample of all specimens and species were included (*n* = 61). Six random samples were analyzed in duplicate and used as quality and reproducibility controls for the protocol. The ddRAD libraries were prepared following a previously published protocol (Daguin-Thiébaut et al. 2021). The libraries were sequenced in 75-bp paired-end reads using the Illumina NextSeq 550 Sequencing System. Raw reads were processed using the software pipeline STACKS v. 1.44 (Catchen et al. 2011) for demultiplexing, quality filtering, *de novo* locus assembly, and SNP screening. The process_radtags module was used to remove barcodes and to discard low-quality reads (Catchen et al. 2011). The ustacks module was used to merge filtered RADtags into loci within each specimen after an evaluation and selection of the best combination of parameters (-m 2, -M 2, -n 2), following the recommendations and guidelines of previous studies (Paris et al. 2017; Rochette and Catchen 2017). Only loci genotyped in at least 80% of individuals (−r 0.8) were considered. The SNPs obtained after *de novo* locus assembly were filtered for some parameters with TASSEL v. 5. Namely, loci with heterozygosity > 0.8 and a minor allele frequency (MAF) < 5% were excluded.

A total of 3692 individual multilocus genotypes obtained from ddRADseq were used to group specimens according to their SNP profiles. Specimens were clustered according to the total genetic variation using a Principal Coordinate Analysis (PCoA) conducted in GENEALEX v. 6.5 (Peakall and Smouse 2012). The most likely number of genetic clusters using a Bayesian clustering approach was obtained using the program STRUCTURE v. 2.1 (Pritchard et al. 2000). The model of correlated allele frequencies and admixture ancestry without sampling localities as priors was used. The software run consisted of ten replicates for each *K* value from 1 to 4 with 1,000,000 Markov chain Monte Carlo (MCMC) generations after a burn-in of 100 000 iterations. The results of the assignment probabilities of individuals (*q*) to each cluster were obtained through the CLUMPAK server (Kopelman et al. 2015), considering an MCL threshold for similarity scores of 0.90. The optimal *K* value was inferred with STRUCTURE HARVESTER using the Evanno method (Evanno et al. 2005; Earl and von Holdt 2012). We assessed pairwise genetic differentiation between the clusters identified using the *F_ST_* estimator of Weir and Cockerham (1984). The *F_ST_* values were estimated using the analysis of molecular variance (AMOVA) with 9999 permutations, as implemented in GENALEX v. 6.5.

## ﻿Results

### ﻿Morphological data

**Figure 2.**
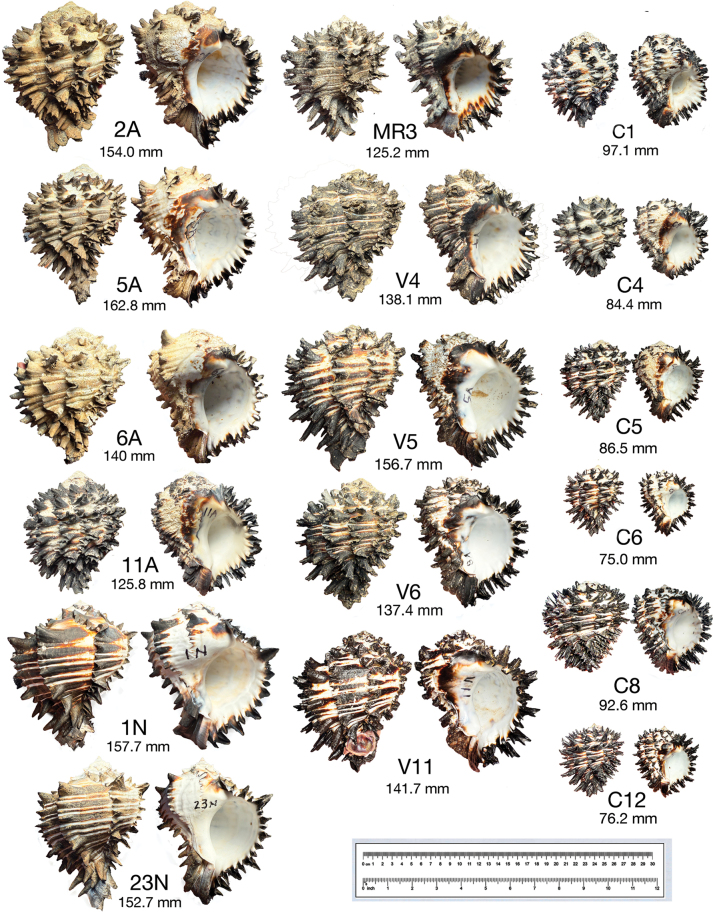
Dorsal and ventral views of shells of *M.nigritus* (1N and 23N), *M.radix* (2A, 6A, V5, C1 and C4), *M.ambiguus* (5A, 12A, MR3, V6, C5, C6, C8, C12) and individuals that are difficult to classify *M.radix*/*ambiguus* (V4 and V11). The measurements given are shell lengths. More specimens and different views of the shells are presented in Suppl. material 2: figs S1–S4.

The 80 *Muricanthus* specimens were classified based on photographs and morphological descriptions published by Houart and Wiedrick (2021). Twenty-five individuals were assigned to *M.nigritus*, 14 were identified as *M.radix*, 32 were classified as *M.ambiguus*, and nine were identified as *M.radix/ambiguus* (Suppl. material 1). These nine specimens had morphological characteristics common to *M.radix* and *M.ambiguus*, making it impossible to assign them to a specific species. Examples of the different species and specimens where correct taxonomic identification is difficult are illustrated in Fig. 2. The morphometric data and identification outcomes of all collected *Muricanthus* specimens are reported in Suppl. material 1. Photographs of six representative examples of specimens for each of the three geographical areas are shown in Suppl. material 2: figs S1, S2 (Magdalena Bay, Baja Sur, Mexico), Suppl. material 2: fig. S3 (Jalisco, Mexico) and Suppl. material 2: fig. S4 (Isla Cebaco, Panama).

### ﻿Haplotype networks and phylogenetic inferences

**Figure 3.**
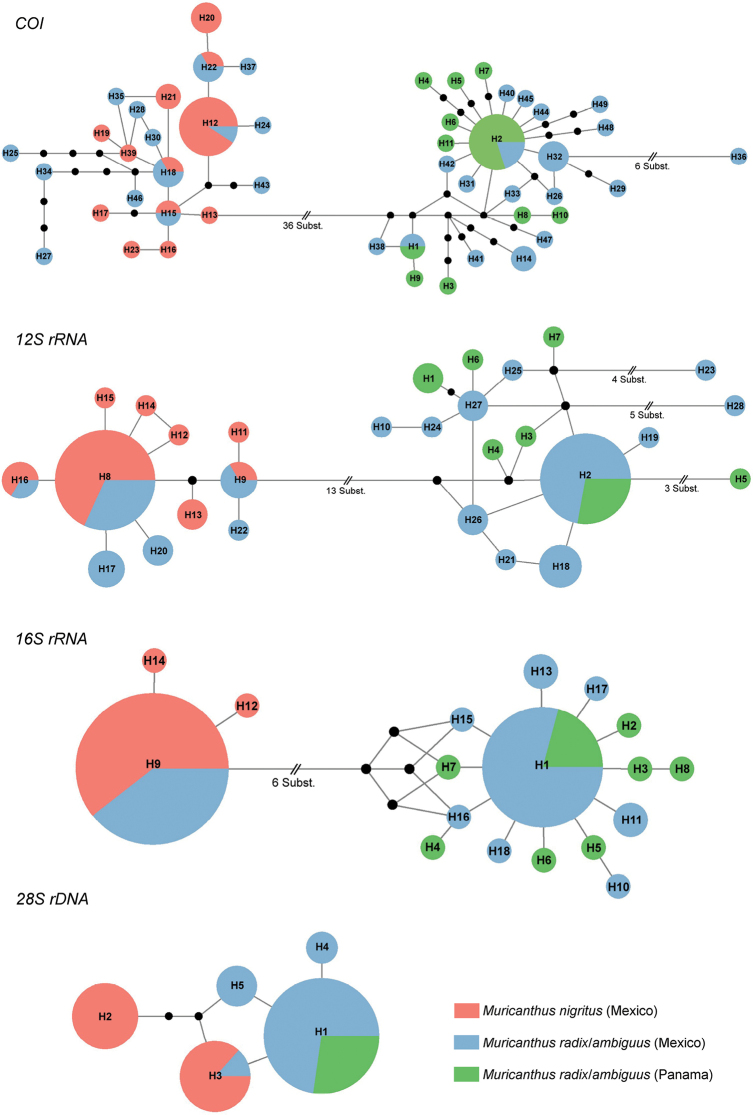
Median-joining networks of all haplotypes identified for the four gene regions studied. Haplotypes are represented by different circles with a size proportional to their frequency (see Suppl. material 1). All cases in which more than one nucleotide substitution occurs between haplotypes are indicated, and all other haplotypes are separated by a single nucleotide change. Solid black circles represent unsampled haplotypes.

**Figure 4.**
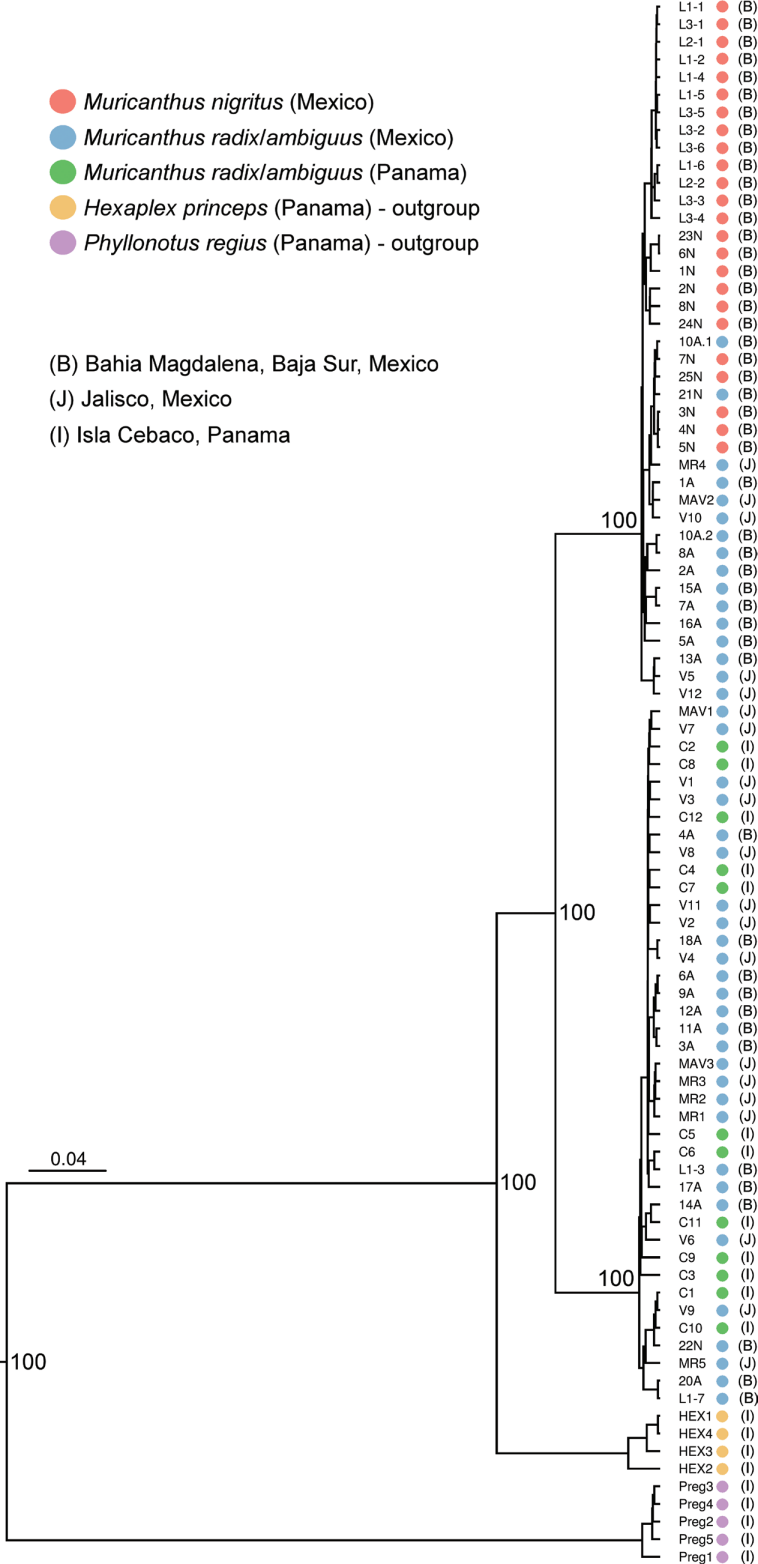
Bayesian maximum clade credibility tree of the concatenated sequences (*COI*, *12S rRNA*, *16S rRNA*, *28S rDNA*). Posterior probabilities for the nodes of the most divergent clades are presented.

The partial sequences of mtDNA *COI* (662 bp), *12S rRNA* (approx. 560 bp), *16S rRNA* (532 bp) and nuclear *28S rDNA* (approx. 1430 bp) were successfully sequenced for the 80 *Muricanthus* specimens analyzed. As expected, a high number of haplotypes were identified in the mitochondrial DNA sequences (49 for *COI*, 28 for *12S rRNA*, and 18 for *16S rRNA*) and a smaller number in the *28S rDNA* nuclear sequences (5 haplotypes). The four median-joining statistical parsimony networks revealed that various mtDNA and nuclear haplotypes are shared by specimens of *M.nigritus* and *M.radix*/*ambiguus* collected in Mexico (Fig. 3). Several specimens of *M.radix*/*ambiguus* from Mexico exhibited specific haplotypes of *M.nigritus*; however, none of the *M.nigritus* specimens analyzed contained any of the *M.radix/ambiguus* specific haplotypes identified, considering the species identification determined by shell morphology (Fig. 3; Suppl. material 1). No shared haplotype was detected between *M.nigritus* from Mexico and *M.radix*/*ambiguus* from Panama (Fig. 3). Specimens of *M.radix/ambiguus* from Mexico and Panama also share several haplotypes (Fig. 3). Two major haplogroups are clearly separated in the *COI*, *12S rRNA*, and *16S rRNA* networks, one corresponding to *M.nigritus* and *M.radix*/*ambiguus* from Mexico, and the other corresponding *M.radix*/*ambiguus* from Mexico and Panama. The Bayesian maximum clade credibility tree obtained using concatenated sequences confirmed the existence of two main, well-supported clades: one including *M.nigritus* and *M.radix*/*ambiguus* haplotypes from Mexico, and the other corresponding to *M.radix*/*sambiguus* haplotypes from Mexico and Panama (Fig. 4). The individual trees of the three mtDNA regions analysed show very similar results (Suppl. material 2: figs S5–S7). The *28S rDNA* tree also shows some differentiation, but less pronounced (Suppl. material 2: fig. S8).

### ﻿Species delimitation

The best species partition based on the ASAP-score split our complete *COI* dataset (including the outgroup species) in 4 hypothetical species (threshold distance = 3.88%, Suppl. material 2: fig. S9a). This analysis split the samples of the three *Muricanthus* species into only two hypothetical species. The ASAP analysis of the *COI* dataset of *Muricanthus* samples alone also suggests the presence of only two hypothetical species, according to the best ASAP-score (threshold distance = 3.34%; Suppl. material 2: fig. S9b).

### ﻿Estimation of genetic clusters from ddRADseq dataset

**Figure 5.**
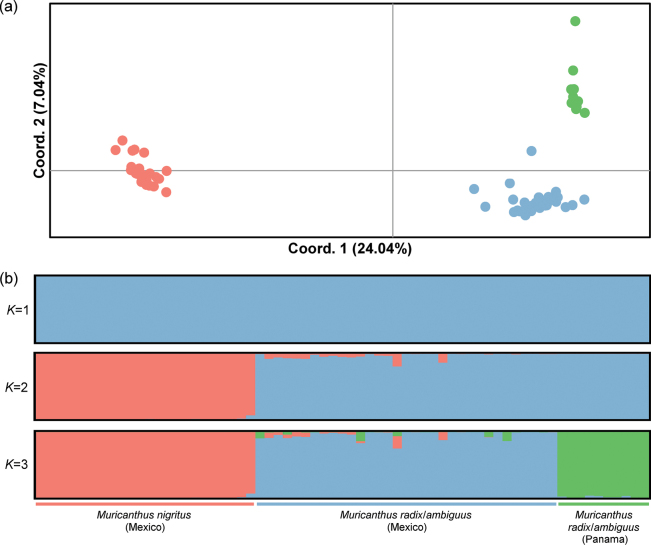
Characterization of genetic clusters obtained using SNP data **a** PCoA plot of genetic distances estimated using the method of Smouse and Peakall (1999)**b** Clusters obtained from the Bayesian clustering analysis without prior information on sample location. Each vertical line corresponds to one individual. The cluster assignments obtained for each *K* tested are represented with different colors, where the optimal number of clusters was estimated for *K* = 2.

The ddRADseq approach was applied to confirm the existence of only two phylogenetic groups among *M.nigritus*, *M.radix*, and *M.ambiguus* through a more in-depth molecular analysis. Our *de novo* assembly with ustacks showed that the number of ddRAD tags recovered from the samples analyzed varied from 119321 to 213181 with a mean coverage of 4×. These ddRAD-tags contained 996260 SNPs. After SNP filtering, the ddRASseq protocol allowed the selection of 3692 SNPs used to cluster the specimens according to their genetic divergence/differentiation (Suppl. material 3). The PCoA and Bayesian clustering approaches showed very similar results (Fig. 5a, b). PCoA separated the *M.nigritus*, *M.radix/ambiguus* (Mexico) and *M.radix/ambiguus* (Panama) (Fig. 5a). The optimal number of clusters inferred in the Bayesian clustering analysis without sample location prior was *K* = 2, corresponding to *M.nigritus* and *M.radix/ambiguus* (Mexico and Panama) (Fig. 5b). However, the simulations for *K* = 3 also made it possible to differentiate *M.radix/ambiguus* (Mexico) and *M.radix/ambiguus* (Panama) (Fig. 5b). It was not possible to differentiate any further groups within these three final clusters. The pairwise genetic differentiation between cluster 1 (*M.nigritus*) and cluster 2 (*M.radix/ambiguus*, Mexico) was *F_ST_* = 0.193, p < 0.001, between cluster 1 and cluster 3 (*M.radix/ambiguus*, Panama) was *F_ST_* = 0.242, p < 0.001, and between cluster 2 and cluster 3 was *F_ST_* = 0.107, p < 0.001. The multilocus genotype data clearly differentiated the *M.nigritus* specimens from the *M.radix/ambiguus* specimens. None of the molecular techniques applied in this work made it possible to differentiate specimens identified as *M.radix* and *M.ambiguus* based on morphology.

### ﻿Nomenclatural act

*Muricanthusambiguus* (A.L. Reeve, 1845) is here synonymized with *Muricanthusradix* (Gmelin, 1791) considering the lack of genetic differentiation between the morphologically assigned specimens.

## ﻿Discussion

The genus *Muricanthus* includes some species that have undergone several taxonomic reclassifications over the last few centuries (Houart and Wiedrick 2021). The difficulties in the proper taxonomic classification of *M.radix* and *M.ambiguus* have been recognized for many years, most recently by Pittman (2023), who pointed out that it is not possible to accurately assign specimens due to the considerable morphological variation seen in their shell structure. Their study hypothesizes that *M.ambiguus* may be a transitional form of *M.radix* from the south and *M.nigritus* from the north (Pittman 2023). The possibility that *M.nigritus* and *M.ambiguus* are northern subspecies of *M.radix* is also highlighted in some publications (Fischer et al. 1995; Escamilla-Montes et al. 2018). *Muricanthusnigritus* is the easiest to identify taxonomically due to its horn-shaped spines, with the spines on the mid-section of the last whorl being shorter than those on other sections and absent in *M.radix* or *M.ambiguus*. *Muricanthusnigritus* also has a very short or missing labral tooth (Houart and Wiedrick 2021; Iwamasa 2023). In the case of specimens of *M.radix* and *M.ambiguus*, the spines are nearly identical and flowery, and both also have a noticeable labral tooth (Houart and Wiedrick 2021). There are differences in spire height, siphonal canal length, and the number of varices, but the variations in morphological structure among the specimens make it difficult to assign some to either species. While there are distinct examples of specimens that can be classified taxonomically as either *M.radix* or *M.ambiguus*, a larger sample of the species reveals difficulties in their assignment, as shown by the numerous examples reported in this study. These species are also commonly misclassified in online biodiversity databases (GBIF Secretariat 2023a, b, c). The high diversity of shell shapes within various mollusk taxa has raised several questions about the possible overestimation or underestimation of the number of species in the classifications based solely on shell morphometrics (Shea et al. 2011). It is well known that environmental and genetic factors both affect shell structure (Gemmell et al. 2018; Clark et al. 2020; Iwamasa 2023). The *Muricanthus* specimens collected from the three different geographical areas showed differences in size, shape, number of varices, and condition, likely due to the environmental conditions in which they developed. From the earliest stages of veliger formation to juvenile and adult stages, the shell continues to grow volumetrically and can produce new varices (Cudney-Bueno et al. 2008; Iwamasa 2023). Environmental conditions can affect the morphology of the shell, resulting in similar-looking specimens between different species and regions. In addition, we observed that many adult shells also show signs of erosion due to predation and aging, further complicating identification. Given the subjectivity and difficulties in the species identification of several *Muricanthus* specimens, an integrative approach with the inclusion of several molecular markers can be an effective strategy to address these issues (Nocella et al. 2024).

The analysis of variations in mitochondrial and nuclear DNA has helped to clarify various taxonomic ambiguities in a wide diversity of mollusk taxa (Xu et al. 2024). In our study, the analysis of three mitochondrial sequences (*COI*, *12S rRNA*, and *16S rRNA*) clearly differentiated the specimens into two groups. Interestingly, one of the groups included individuals of *M.nigritus* (Mexico) and *M.radix/ambiguus* (Mexico) and another included individuals of *M.ambiguus* and *M.radix* (Mexico and Panama). The analysis of the nuclear *28S rDNA* sequences revealed similar results, although the differentiation was lower. This is expected, as the mutation rates of nuclear DNA sequences are lower than those of mitochondrial DNA (Duda 2021). The fact that several haplotypes are shared between *M.nigritus* and *M.radix/ambiguus* may indicate the possible occurrence of historical and/or recent hybridization between these species. Our data suggest a potential asymmetrical hybridization process in the sympatric areas, since only the *M.ambiguus/radix* species presented *M.nigritus* haplotypes. Mitochondrial DNA introgression has been reported in several organisms, including mollusk species (e.g., Toews and Brelsford 2012; Yu et al. 2014; Garcia-Souto et al. 2024). Introgressive hybridization is an evolutionary process involving different biological processes, such as adaptation and response to environmental changes, speciation, and regulation of biodiversity levels (Dowling and Secor 1997).

None of the “classical” mitochondrial and nuclear DNA markers differentiated *M.radix* from *M.ambiguus*, highlighting the presence of several common haplotypes between the specimens from Mexico and Panama. This does not support the classification of *M.radix* and *M.ambiguus* as two distinct species based on morphometric data, although the sharing of mtDNA haplotypes can happen in some cases when very recent evolutionary processes of species divergence are involved, or where ancestral mtDNA polymorphisms are retained in recently diverged clades (Moritz et al. 1987; Galtier et al. 2009). The DNA-based species delimitation analyses support the data obtained from the Bayesian phylogenetic inferences, highlighting that the *Muricanthus* samples are split into only two species.

In this context, double digest restriction-site associated DNA sequencing (ddRAD-seq) was used for a more in-depth analysis of the genetic differentiation among the *Muricanthus* individuals. Several studies on species classification have already shown that this methodology is an efficient approach to clarify doubts left by “classic” DNA markers (e.g., Glon et al. 2021; Kim and Roe 2021; Obiol et al. 2023). The principal component and Bayesian clustering analysis of the new SNPs discovered in this study showed very similar results, allowing the specimens to be grouped into three clusters. Specimens of *M.nigritus* were clearly differentiated from specimens of *M.radix*/*ambiguus*. It was also possible to differentiate specimens of *M.radix*/*ambiguus* (Mexico) from specimens of *M.radix/ambiguus* (Panama) in both analyses. Once again, it was not possible to differentiate any cluster/individual between *M.radix* and *M.ambiguus*. Furthermore, Bayesian analysis suggests that the optimal number of clusters is *K* = 2, where *M.radix* (Mexico and Panama) and *M.ambiguus* (Mexico and Panama) are part of a single cluster. This finding indicates that the genetic differentiation observed between *M.radix*/*M.ambiguus* from Panama and Mexico may be associated with the geographical distance between these populations. Limitations in the dispersal potential between distant geographical areas and/or discontinuity of suitable habitats can explain the genetic differentiation observed. Although the analysis of the genetic data did not allow us to differentiate between *M.radix* and *M.ambiguus* in Mexico or in Panama, we noticed that the shells of the Panama specimens are generally smaller, at least in this collection site. These variations in shell size may be caused by genetic or environmental factors. Variations in shell size in the same gastropod species between different regions have been reported in several studies (e.g., Irie 2006; Cudney-Bueno et al. 2008; Malve et al. 2018; Matos et al. 2020; Iwamasa 2023). Within this context, our study also raises the question of whether *M.callidinus* Berry, 1958 and *M.strausi* Verrill, 1950 are truly distinct and different *Muricanthus* species. The inclusion of molecular data for the proper classification of these species can be an effective method of resolving taxonomic issues and clarifying their phylogenetic positions among the *Muricanthus* species complex.

## ﻿Conclusions

This is the first study to integrate molecular techniques into the process of identifying *Muricanthus* specimens. Our molecular analysis reveals that *M.radix* and *M.ambiguus* are not genetically distinct, despite being classified as separate species based on morphology. In addition, a possible occurrence of mtDNA introgression was observed between *M.nigritus* and *M.radix*/*ambiguus*, suggesting possible hybridization processes between these species. The morphology of all the *Muricanthus* specimens studied from the three largely separated areas showed the problems of species identification based on the morphometric descriptions derived by the original authors and many others since then. The molecular approaches applied in this study highlighted that a morphology-based classification alone may be erroneous in this species complex. The data presented in this research indicate that *M.ambiguus* is a synonym of *M.radix*. Although it is possible to differentiate the *M.nigritus* specimens studied at the morphological and genetic level from *M.radix*/*ambiguus*, further research of more specimens from different regions is needed to clarify the possible hybridization between *M.nigritus* and *M.radix*/*ambiguus*, and the species boundaries. The high level of phenotypic plasticity in *Muricanthus* species is evidenced in this work and in previous studies (Iwamasa 2023; Pittman 2023), which highlights the importance of using an integrative taxonomy approach in future studies on this subject. Our study indicates the need to develop further genetic studies not only to clarify phylogenetic relationships between other *Muricanthus* species, but also to understand their diversity, genetic structure, evolutionary processes, and species distribution. This is crucial for implementing targeted conservation strategies for these species and their habitats, as they are exposed to several anthropogenic pressures.

## ﻿Additional information

### Author ORCIDs

Francisco Morinha https://orcid.org/0000-0003-0621-9591

Ana M. Rocha https://orcid.org/0000-0003-1512-3188

### Data availability

All of the data that support the findings of this study are available in the main text or Supplementary Information.

## Supplementary materials

Supplementary material 1 Database of all the specimens included in this study Data type xlsx Explanation note Sample ID, collection date, country, region, site, approximate coordinates, morphometric parameters (weight, length, width and number of varices), species classification based on shell morphology, structure clusters (from ddRAD data), haplotypes (*COI*, *12S rRNA*, *16S rRNA*, and *28S rDNA*) and GenBank accession numbers for all sequences are reported. https://binary.pensoft.net/file/1340303This dataset is made available under the Open Database License (http://opendatacommons.org/licenses/odbl/1.0/). The Open Database License (ODbL) is a license agreement intended to allow users to freely share, modify, and use this Dataset while maintaining this same freedom for others, provided that the original source and author(s) are credited.Francisco Morinha, James Ernest, Ana Archer-Taveira, Ana M. Rocha, Robert T. Iwamasa

Supplementary material 2 Additional images Data type pdf Explanation note **fig. S1.** Dorsal, ventral, anterior, posterior, left and right side views of six *Muricanthusnigritus* (N) shells from Magdalena Bay, Baja Sur, Mexico. The sample ID is shown to the left of each individual’s panel. Weight (g), length (mm), and number varices are shown. The specimen dimensions are not to scale. See Suppl. material 1 for more information about the species. **fig. S2** Dorsal, ventral, anterior, posterior, left and right side views of six *Muricanthusradix* (R) and *Muricanthusambiguus* (A) shells from Magdalena Bay, Baja Sur, Mexico. The sample ID is shown to the left of each individual’s panel. Weight (g), length (mm) and number varices are shown. The specimen dimensions are not to scale. See Suppl. material 1 for more information about the species. **fig. S3.** Dorsal, ventral, anterior, posterior, left and right side views of six *Muricanthusradix* (R) and *Muricanthusambiguus* (A) shells from Jalisco, Mexico. The sample ID is shown to the left of each individual’s panel. Weight (g), length (mm) and number varices are shown. The specimen dimensions are not to scale. See Suppl. material 1 for more information about the species. **fig. S4.** Dorsal, ventral, anterior, posterior, left and right side views of six *Muricanthusradix* (R) and *Muricanthusambiguus* (A) shells from Isla Cebaco, Panama. The sample ID is shown to the left of each individual’s panel. Weight (g), length (mm) and number varices are shown. The specimen dimensions are not to scale. See Suppl. material 1 for more information about the species. **fig. S5.** Bayesian maximum clade credibility tree of the *COI* sequences. Posterior probabilities for the nodes of the most divergent clades are presented. **fig. S6.** Bayesian maximum clade credibility tree of the *12S rRNA* sequences. Posterior probabilities for the nodes of the most divergent clades are presented. **fig. S7.** Bayesian maximum clade credibility tree of the *16S rRNA* sequences. Posterior probabilities for the nodes of the most divergent clades are presented. **fig. S8.** Bayesian maximum clade credibility tree of the *28S rDNA* sequences. Posterior probabilities for the nodes of the most divergent clades are presented. **fig. S9.** Results of the ASAP analysis using the *COI* datasets. (a) ASAP results from *COI* dataset of all sequences obtained in this study (including outgroup species). (b) ASAP results using only *COI* sequences from *Muricanthus* specimens. The lower the ASAP score, the better the partition (Nb subsets = number of hypothetical species predicted). https://binary.pensoft.net/file/1340304This dataset is made available under the Open Database License (http://opendatacommons.org/licenses/odbl/1.0/). The Open Database License (ODbL) is a license agreement intended to allow users to freely share, modify, and use this Dataset while maintaining this same freedom for others, provided that the original source and author(s) are credited.Robert T. Iwamasa, Francisco Morinha

Supplementary material 3 SNP data Data type vcf Explanation note VCF file with the 3692 SNPs selected after ddRAD-seq analysis. https://binary.pensoft.net/file/1340305This dataset is made available under the Open Database License (http://opendatacommons.org/licenses/odbl/1.0/). The Open Database License (ODbL) is a license agreement intended to allow users to freely share, modify, and use this Dataset while maintaining this same freedom for others, provided that the original source and author(s) are credited.Francisco Morinha, James Ernest, Ana Archer-Taveira, Ana M. Rocha, Robert T. Iwamasa
