# Supplementary material for: ﻿Molecular and morphological data support the synonymy of Muricanthusradix Gmelin, 1791 and Muricanthusambiguus Reeve, 1845 (Gastropoda, Muricidae)
Source: Zookeys. 2025 May 28;1239:281–303. doi: 10.3897/zookeys.1239.143837 (PMC12138812; doi:10.3897/zookeys.1239.143837)

Fig. S1

BAHIA MAGDALENA, BAJA SUR, MEXICO

Muricanthus nigritus

L2-2

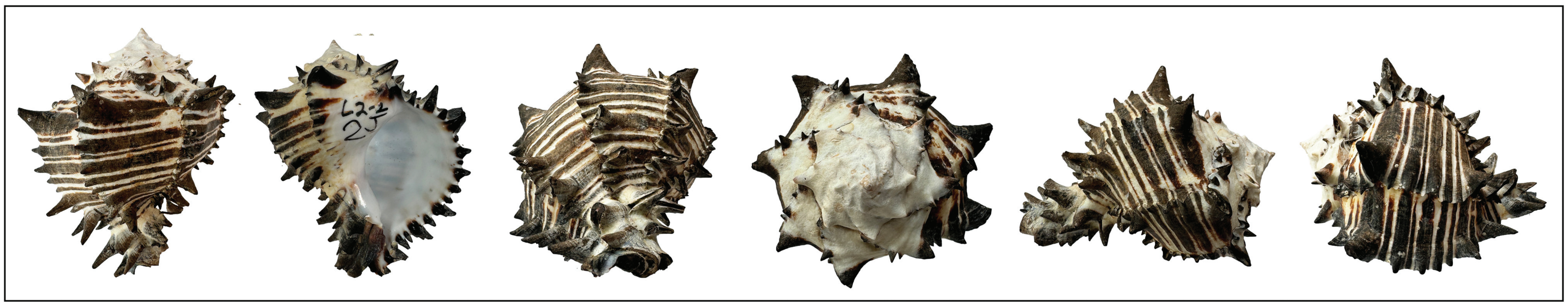

N

245 g 127.9 mm (l) 100.0 mm (w) 7 varices

8N

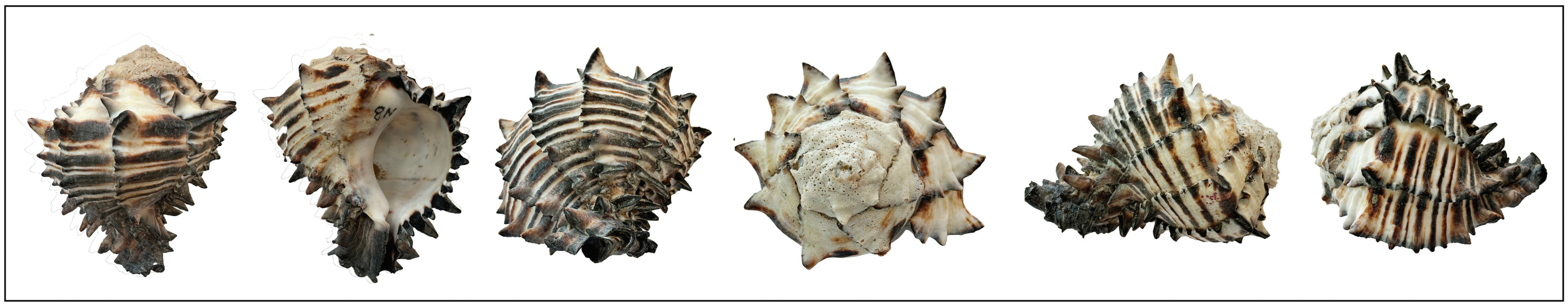

N

502 g 147.1 mm (l) 131.6 mm (w) 10 varices

L2-1

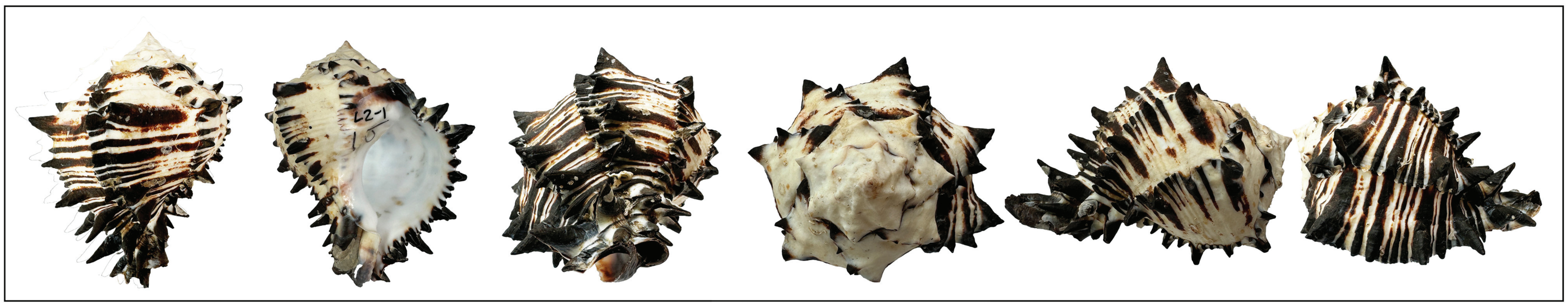

N

336 g 145.2 mm (l) 100.0 mm (w) 7 varices

23N

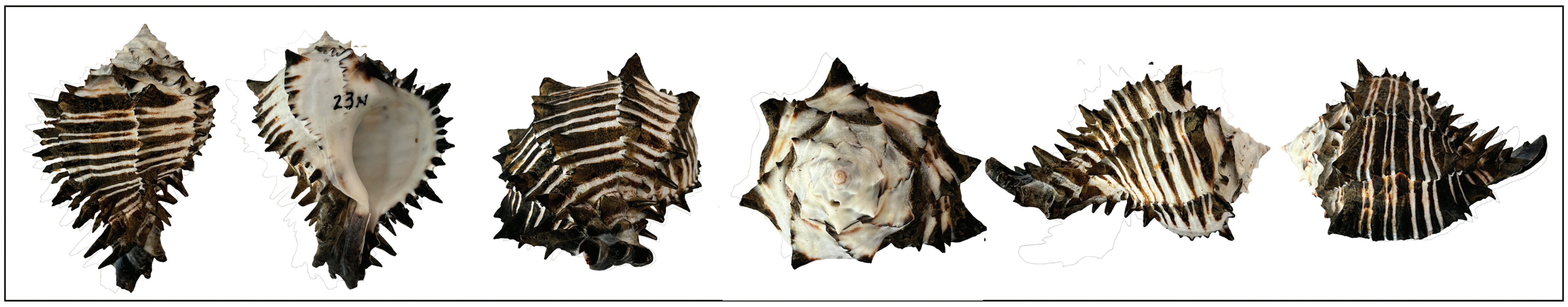

N

369 g 152.7 mm (l) 119.1 mm (w) 8 varices

4N

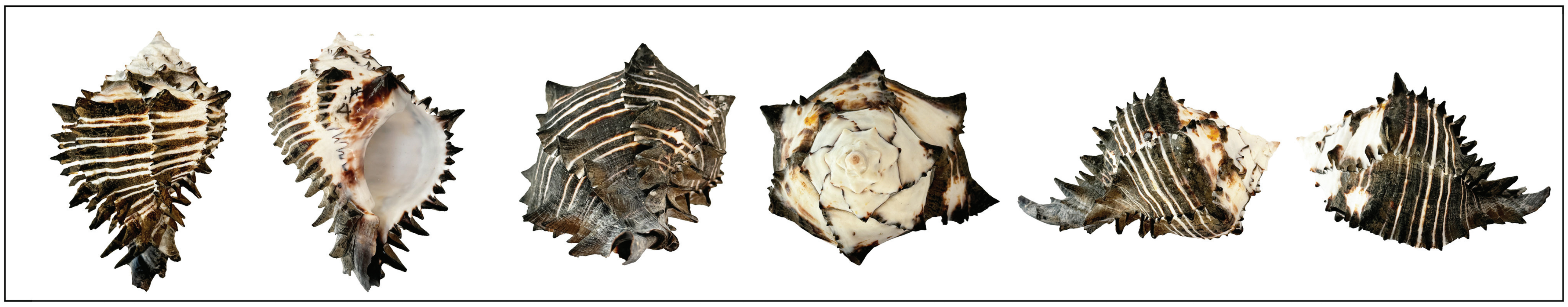

N

308 g 157.2 mm (l) 120.7 mm (w) 6 varices

6N

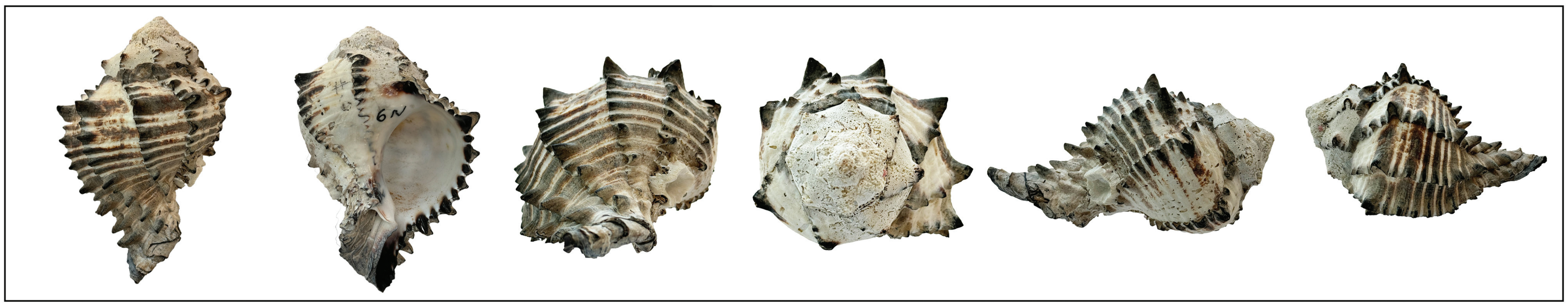

N

462 g 175.7 mm (l) 121.0 mm (w) 8 varices

Fig. S2

BAHIA MAGDALENA, BAJA SUR, MEXICO

Muricanthus radix/Muricanthus ambiguus

11A

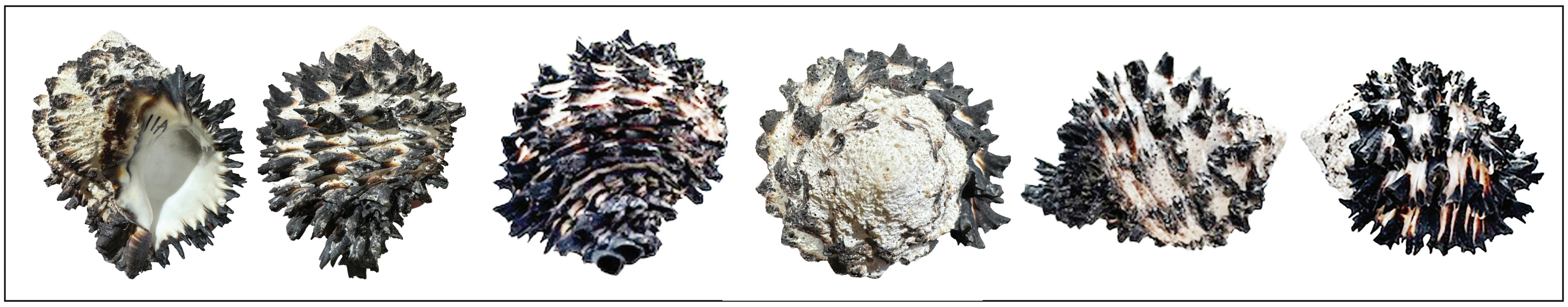

R

429 g 125.8 mm (l) 118.4 mm (w) 12 varices

2A

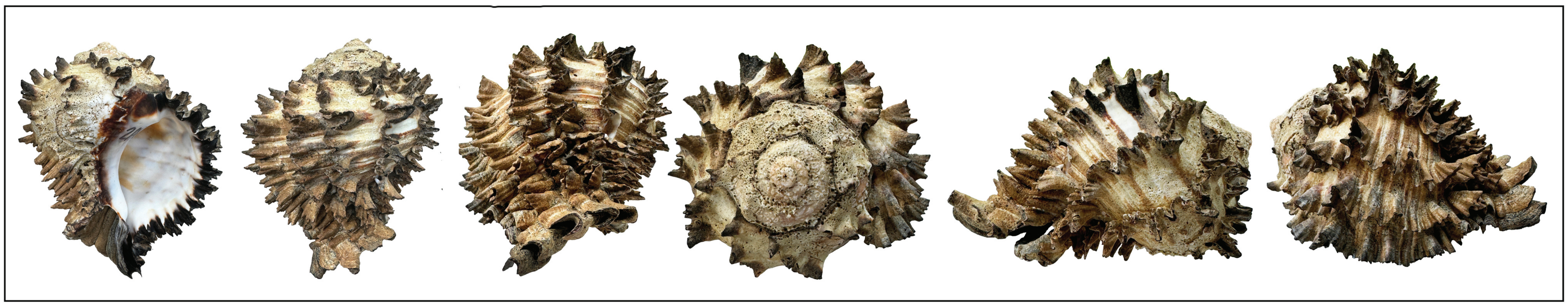

R

442 g 154 mm (l) 126.9 mm (w) 11 varices

1A

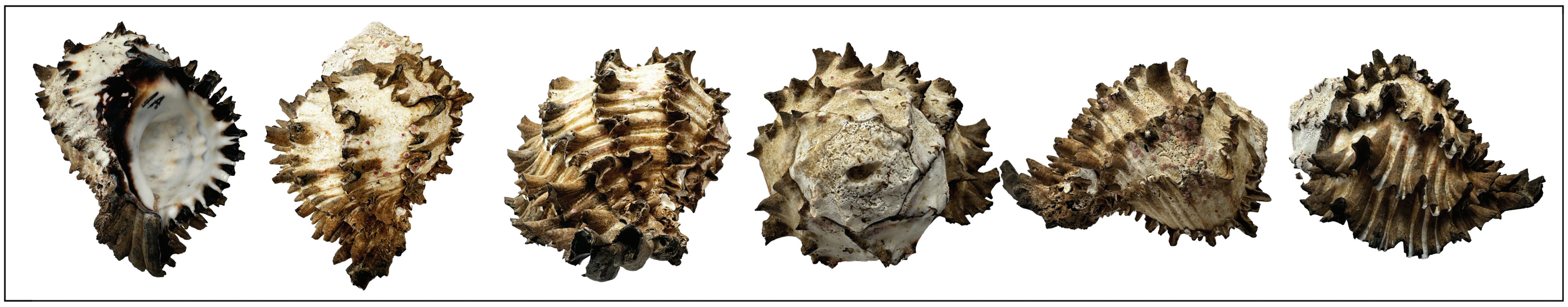

R/A

495 g 159.0 mm (l) 127.5 mm (w) 11 varices

6A

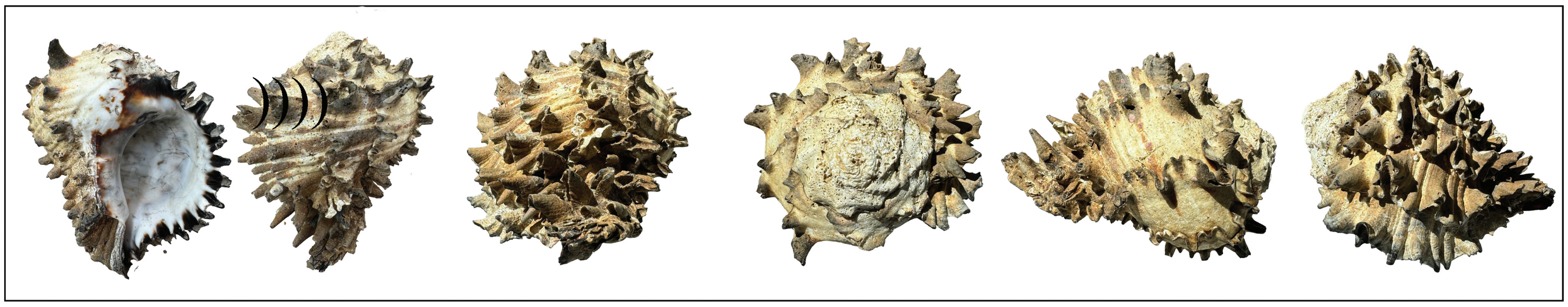

A/R

392 g 140.0 mm (l) 115.3 mm (w) 10 varices

5A

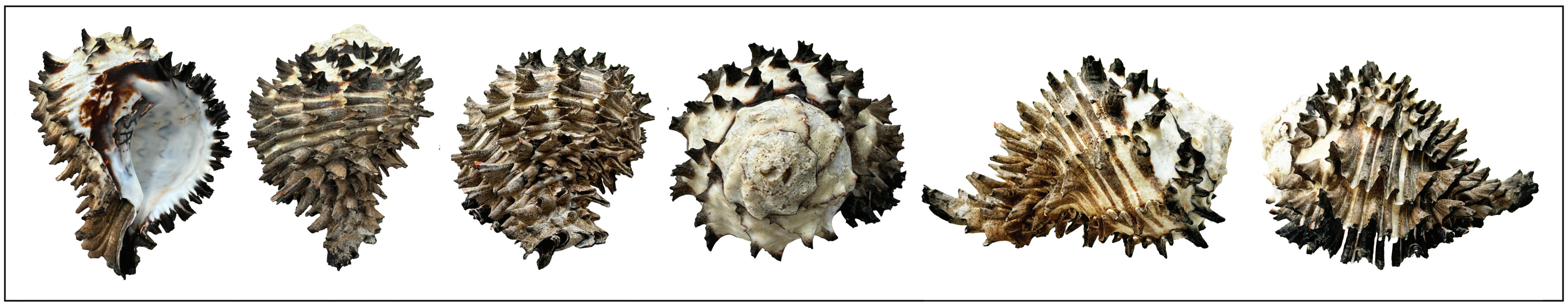

A

431 g 162.8 mm (l) 123.8 mm (w) 12 varices

3A

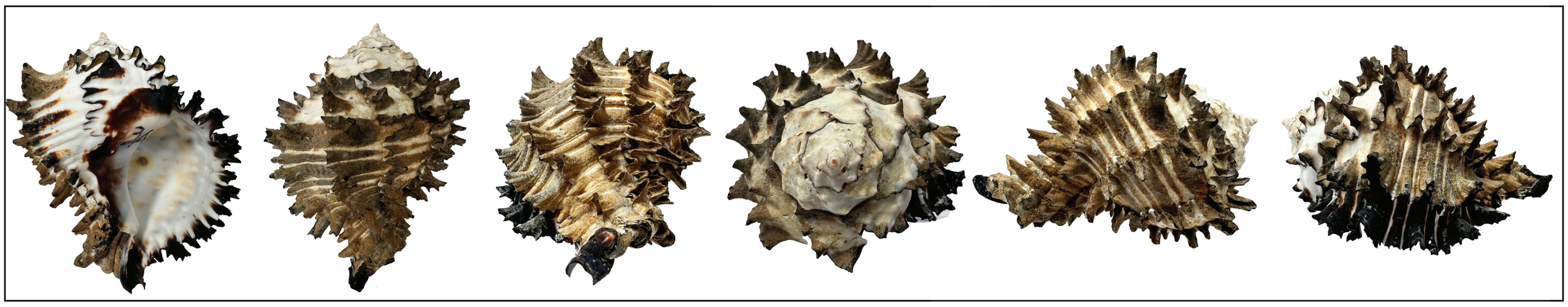

A

301 g 145.9 mm (l) 116.0 mm (w) 11 varices

Fig. S3

JALISCO, MEXICO

Muricannthus radix/Muricanthus ambiguus

MR1

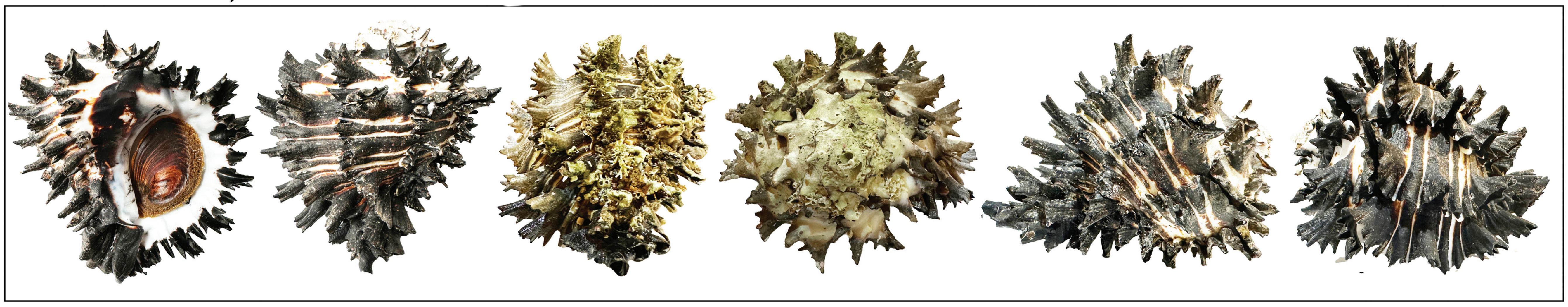

R

481 g 134.9 mm (l) 124.4 mm (w) 10 varices

V4

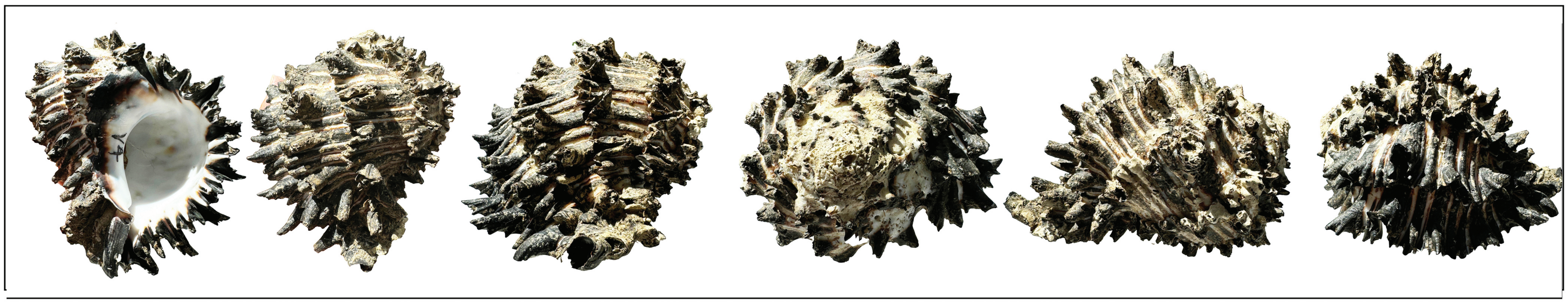

R

519 g 138.1 mm (l) 121.4 mm (w) 10 varices

V5

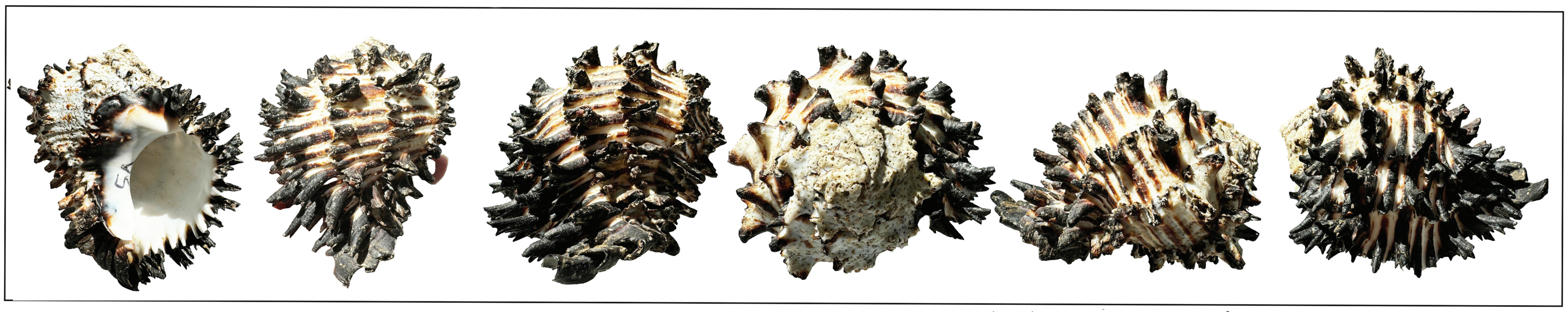

R

502 g 156.7 mm (l) 118.1 mm (w) 11 varices

V6

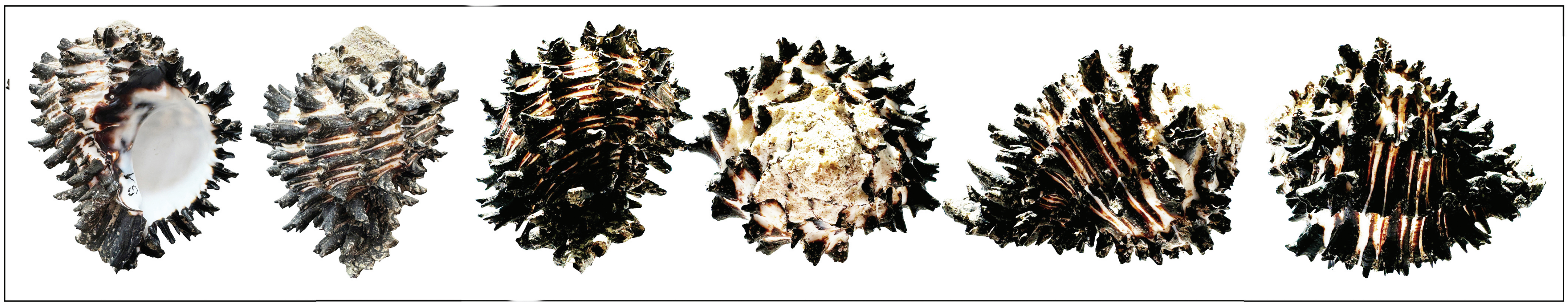

R/A

443 g 137.4 mm (l) 119.8 mm (w) 10 varices

V11

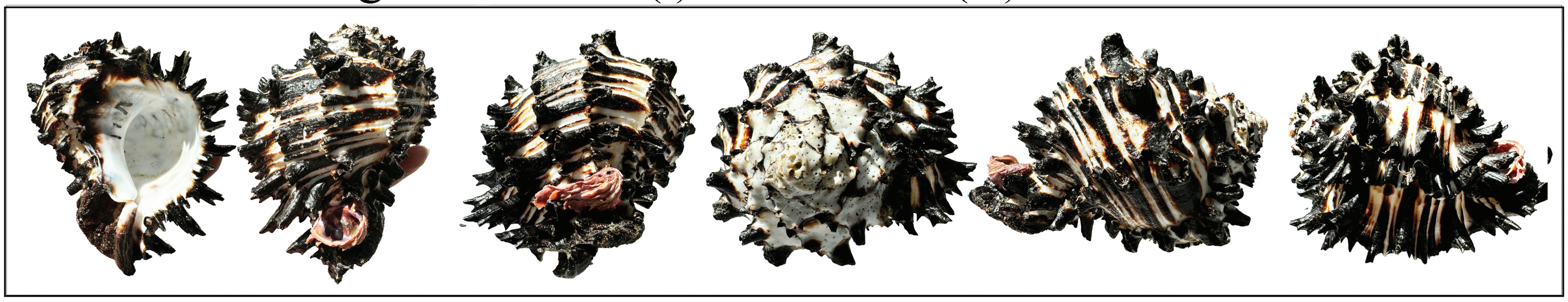

A

431 g 141.7 mm (l) 118.8 mm (w) 10 varices

MR3

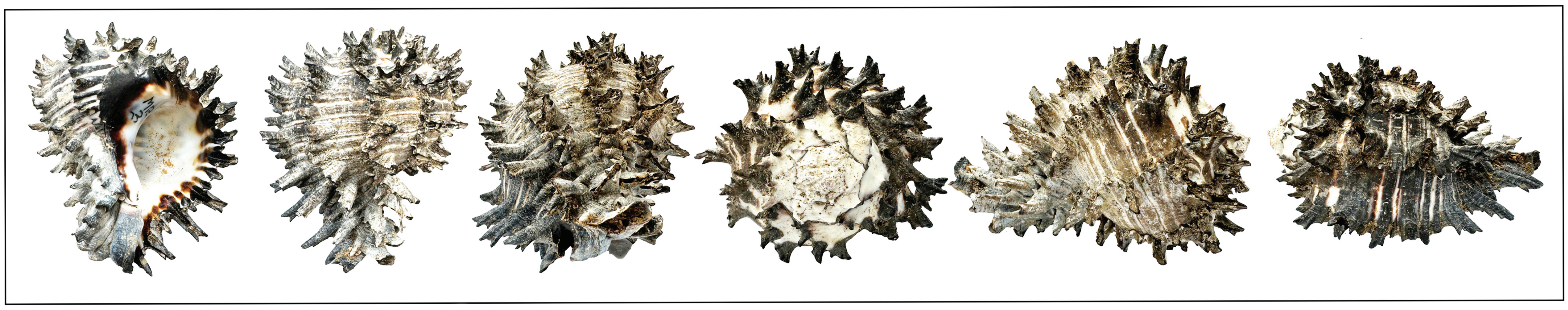

A

293 g 125.2 mm (l) 105.6 mm (w) 9 varices

Fig. S4

ISLA CEBACO, PANAMA

Muricannthus radix/Muricanthus ambiguus

C8

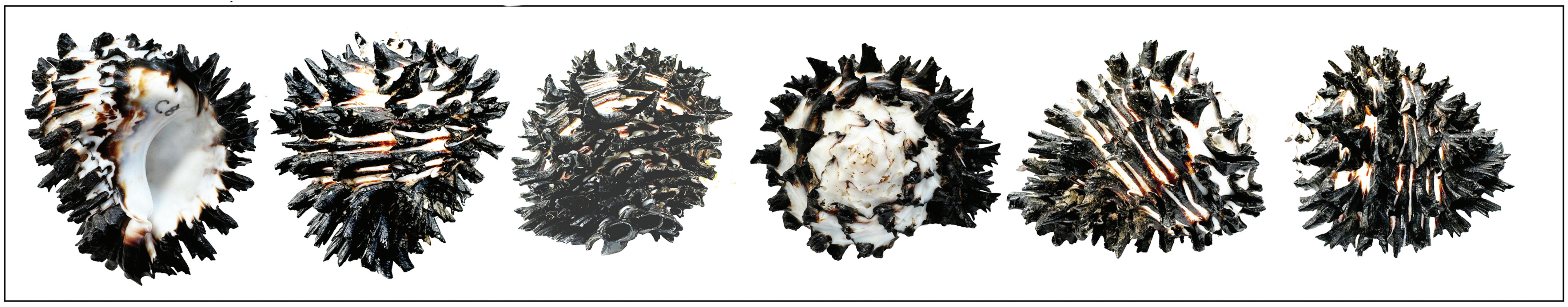

R

239 g   92.6 mm (l)   69.0 mm (w)   12 varices

C12

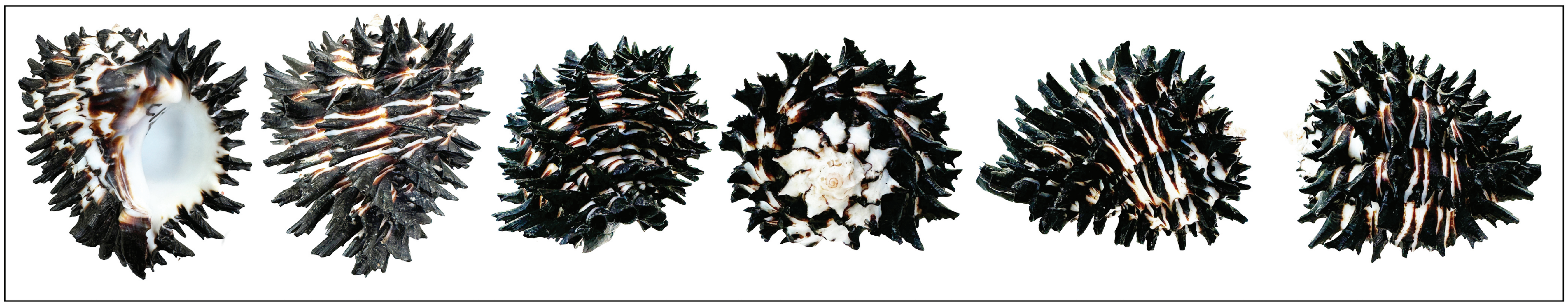

R

125 g   76.2 mm (l)   65.0 mm (w)   11 varices

C4

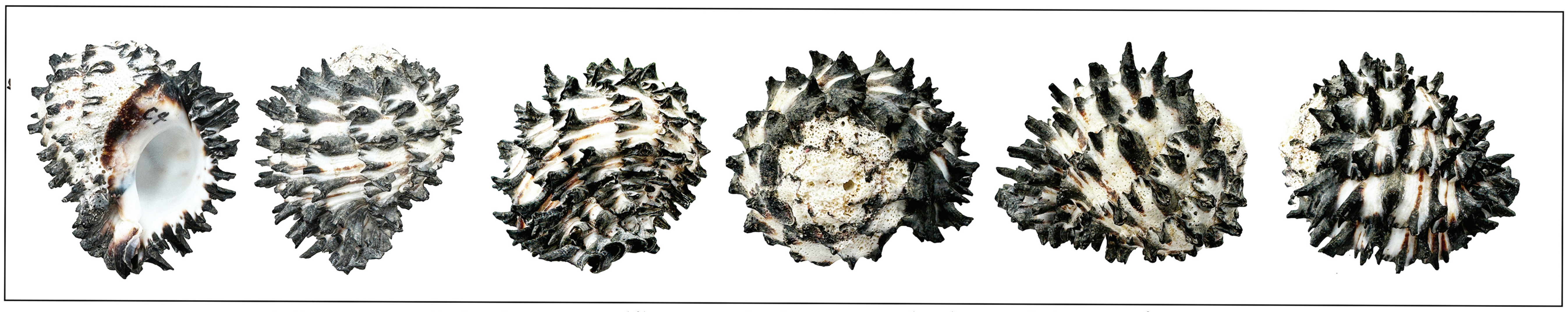

R

195 g   84.4 mm (l)   71.0 mm (w)   11 varices

C1

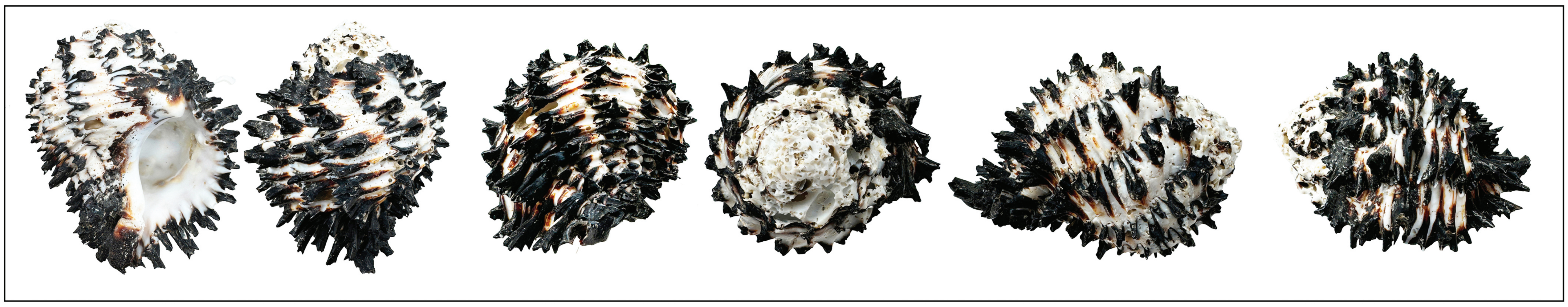

R

221 g   97.1 mm (l)   75.0 mm (w)   12 varices

C5

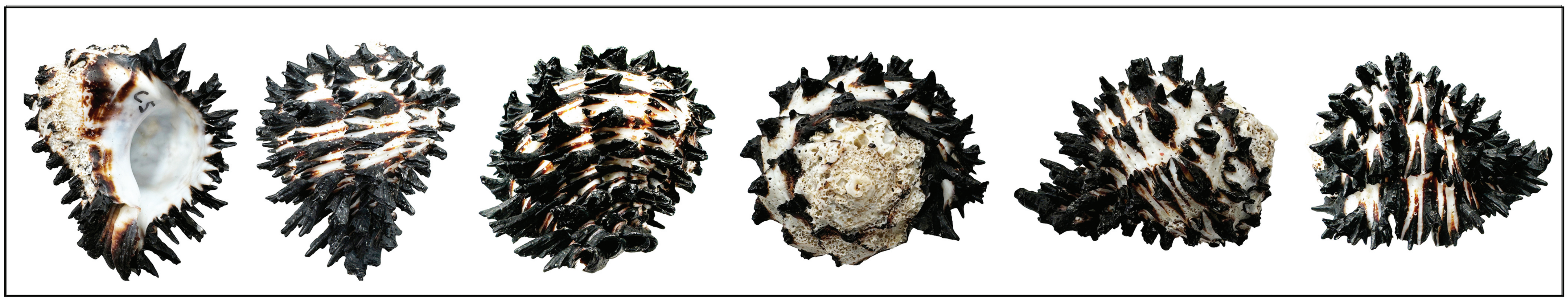

A

114 g   86.5 mm (l)   77.0 mm (w)   10 varices

C6

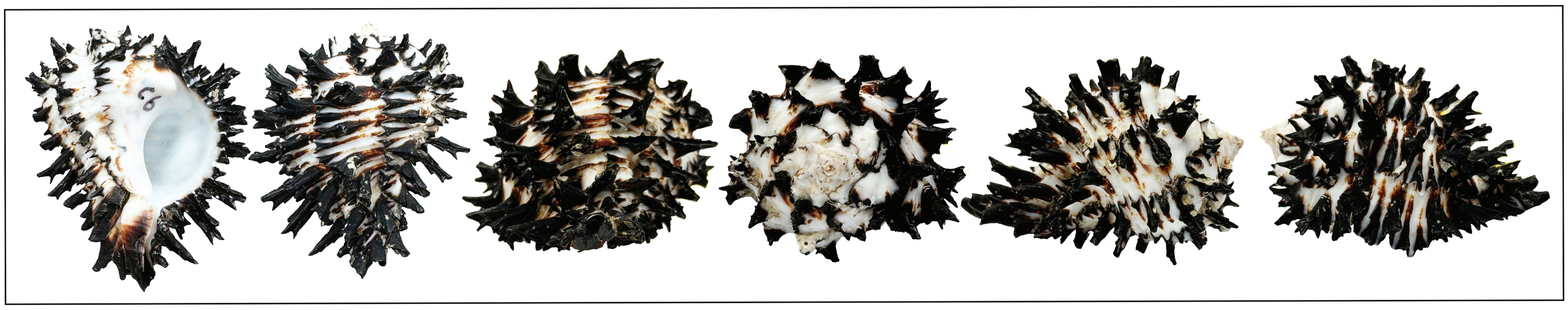

A

89 g   75.0 mm (l)   65.0 mm (w)   9 varices

Fig. S5

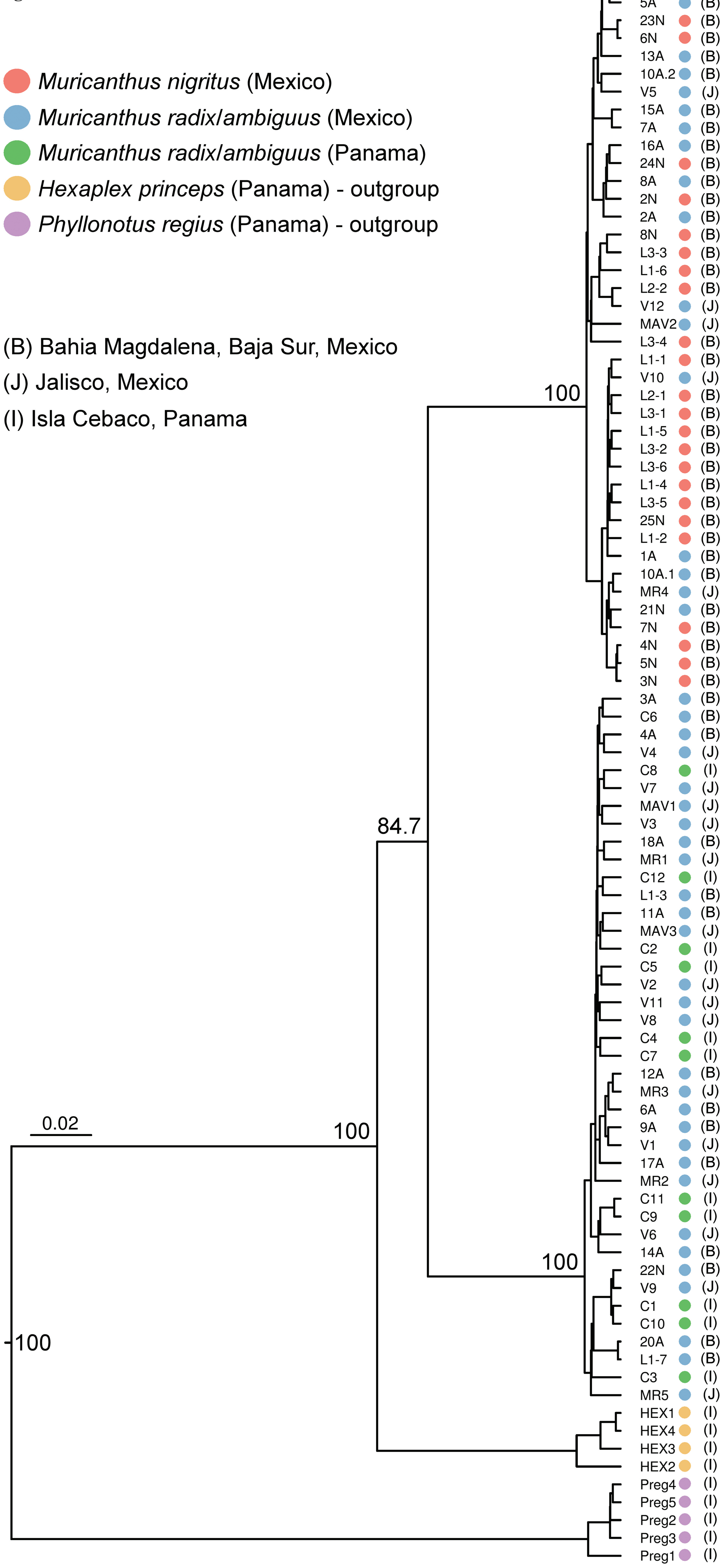

Muricanthus nigritus (Mexico)
Muricanthus radix/ambiguus (Mexico)
Muricanthus radix/ambiguus (Panama)
Hexaplex princeps (Panama) - outgroup
Phyllonotus regius (Panama) - outgroup
(B) Bahia Magdalena, Baja Sur, Mexico
(J) Jalisco, Mexico
(I) Isla Cebaco, Panama
0.02
100
100
84.7
100
100
1N (B)
5A (B)
23N (B)
6N (B)
13A (B)
10A.2 (B)
V5 (J)
15A (B)
7A (B)
16A (B)
24N (B)
8A (B)
2N (B)
2A (B)
8N (B)
L3-3 (B)
L1-6 (B)
L2-2 (B)
V12 (J)
MAV2 (J)
L3-4 (B)
L1-1 (B)
V10 (J)
L2-1 (B)
L3-1 (B)
L1-5 (B)
L3-2 (B)
L3-6 (B)
L1-4 (B)
L3-5 (B)
25N (B)
L1-2 (B)
1A (B)
10A.1 (B)
MR4 (J)
21N (B)
7N (B)
4N (B)
5N (B)
3N (B)
3A (B)
C6 (B)
4A (B)
V4 (J)
C8 (I)
V7 (J)
MAV1 (J)
V3 (J)
18A (B)
MR1 (J)
C12 (I)
L1-3 (B)
11A (B)
MAV3 (J)
C2 (I)
C5 (I)
V2 (J)
V11 (J)
V8 (J)
C4 (I)
C7 (I)
12A (B)
MR3 (J)
6A (B)
9A (B)
V1 (J)
17A (B)
MR2 (J)
C11 (I)
C9 (I)
V6 (J)
14A (B)
22N (B)
V9 (J)
C1 (I)
C10 (I)
20A (B)
L1-7 (B)
C3 (I)
MR5 (J)
HEX1 (I)
HEX4 (I)
HEX3 (I)
HEX2 (I)
Preg4 (I)
Preg5 (I)
Preg2 (I)
Preg3 (I)
Preg1 (I)

Fig. S6

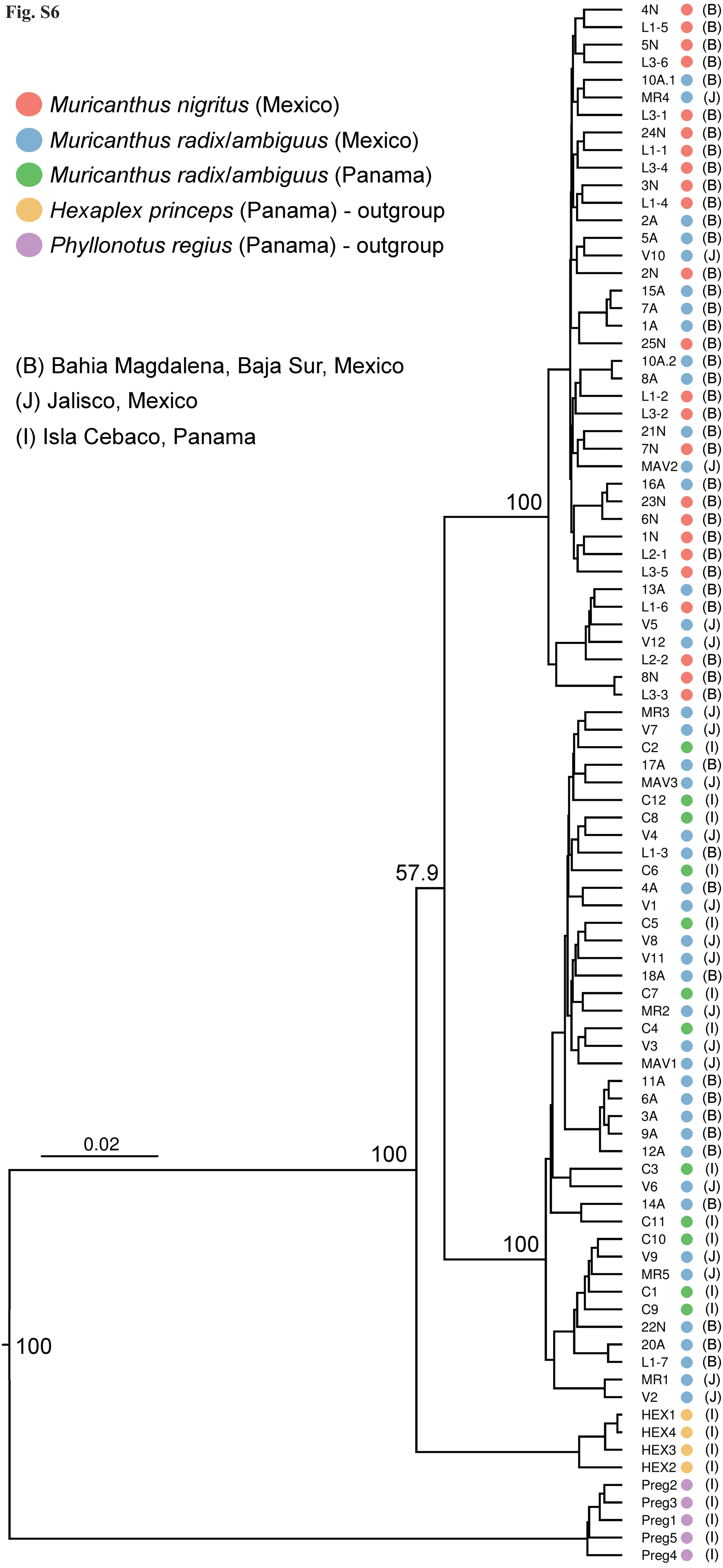
Muricanthus nigritus (Mexico)
Muricanthus radix/ambiguus (Mexico)
Muricanthus radix/ambiguus (Panama)
Hexaplex princeps (Panama) - outgroup
Phyllonotus regius (Panama) - outgroup
(B) Bahia Magdalena, Baja Sur, Mexico
(J) Jalisco, Mexico
(I) Isla Cebaco, Panama
0.02
100
57.9
100
100
100
4N (B)
L1-5 (B)
5N (B)
L3-6 (B)
10A.1 (B)
MR4 (J)
L3-1 (B)
24N (B)
L1-1 (B)
L3-4 (B)
3N (B)
L1-4 (B)
2A (B)
5A (B)
V10 (J)
2N (B)
15A (B)
7A (B)
1A (B)
25N (B)
10A.2 (B)
8A (B)
L1-2 (B)
L3-2 (B)
21N (B)
7N (B)
MAV2 (J)
16A (B)
23N (B)
6N (B)
1N (B)
L2-1 (B)
L3-5 (B)
13A (B)
L1-6 (B)
V5 (J)
V12 (J)
L2-2 (B)
8N (B)
L3-3 (B)
MR3 (J)
V7 (J)
C2 (I)
17A (B)
MAV3 (J)
C12 (I)
C8 (I)
V4 (J)
L1-3 (B)
C6 (I)
4A (B)
V1 (J)
C5 (I)
V8 (J)
V11 (J)
18A (B)
C7 (I)
MR2 (J)
C4 (I)
V3 (J)
MAV1 (J)
11A (B)
6A (B)
3A (B)
9A (B)
12A (B)
C3 (I)
V6 (J)
14A (B)
C11 (I)
C10 (I)
V9 (J)
MR5 (J)
C1 (I)
C9 (I)
22N (B)
20A (B)
L1-7 (B)
MR1 (J)
V2 (J)
HEX1 (I)
HEX4 (I)
HEX3 (I)
HEX2 (I)
Preg2 (I)
Preg3 (I)
Preg1 (I)
Preg5 (I)
Preg4 (I)

Fig. S7

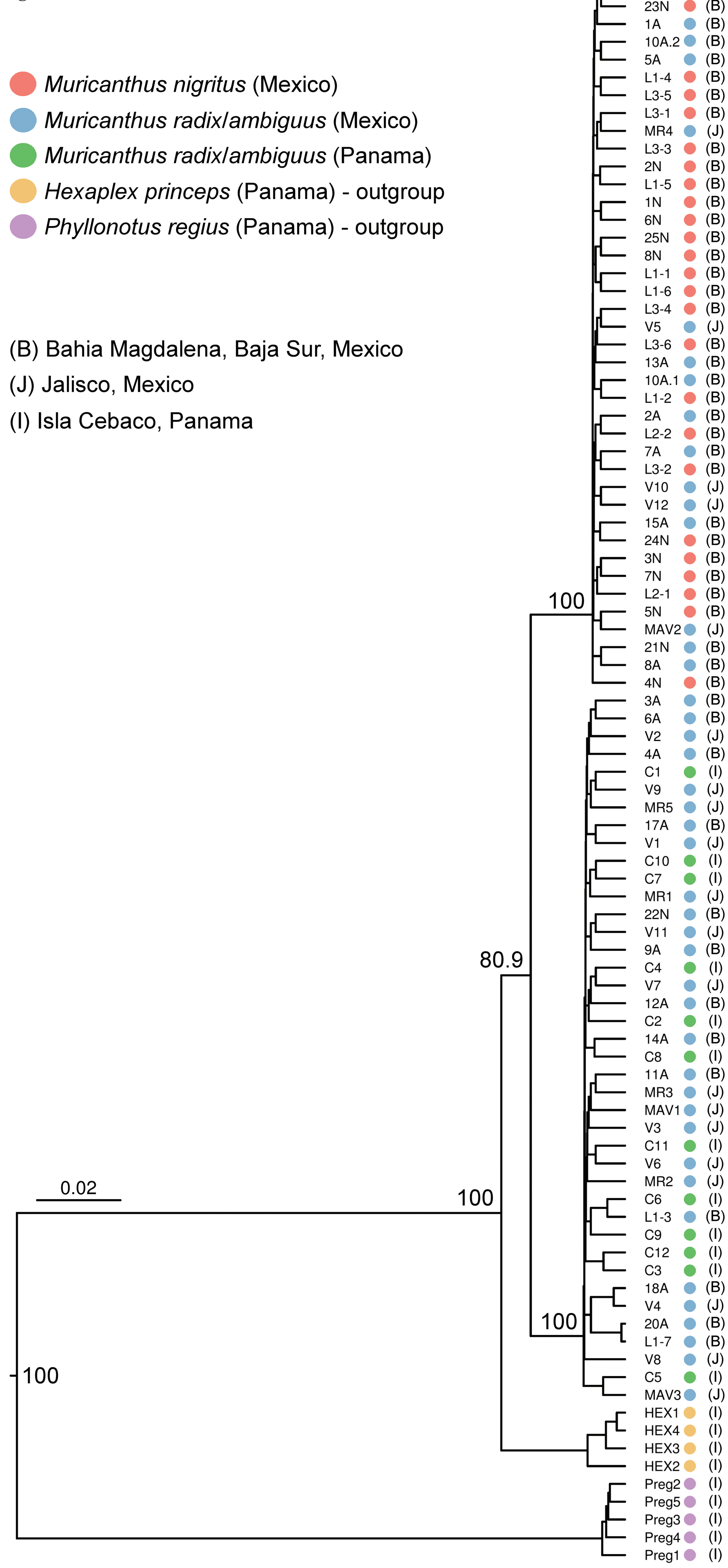
Muricanthus nigritus (Mexico)
Muricanthus radix/ambiguus (Mexico)
Muricanthus radix/ambiguus (Panama)
Hexaplex princeps (Panama) - outgroup
Phyllonotus regius (Panama) - outgroup
(B) Bahia Magdalena, Baja Sur, Mexico
(J) Jalisco, Mexico
(I) Isla Cebaco, Panama
0.02
100
100
80.9
100
100
16A (B)
23N (B)
1A (B)
10A.2 (B)
5A (B)
L1-4 (B)
L3-5 (B)
L3-1 (B)
MR4 (J)
L3-3 (B)
2N (B)
L1-5 (B)
1N (B)
6N (B)
25N (B)
8N (B)
L1-1 (B)
L1-6 (B)
L3-4 (B)
V5 (J)
L3-6 (B)
13A (B)
10A.1 (B)
L1-2 (B)
2A (B)
L2-2 (B)
7A (B)
L3-2 (B)
V10 (J)
V12 (J)
15A (B)
24N (B)
3N (B)
7N (B)
L2-1 (B)
5N (B)
MAV2 (J)
21N (B)
8A (B)
4N (B)
3A (B)
6A (B)
V2 (J)
4A (B)
C1 (I)
V9 (J)
MR5 (J)
17A (B)
V1 (J)
C10 (I)
C7 (I)
MR1 (J)
22N (B)
V11 (J)
9A (B)
C4 (I)
V7 (J)
12A (B)
C2 (I)
14A (B)
C8 (I)
11A (B)
MR3 (J)
MAV1 (J)
V3 (J)
C11 (I)
V6 (J)
MR2 (J)
C6 (I)
L1-3 (B)
C9 (I)
C12 (I)
C3 (I)
18A (B)
V4 (J)
20A (B)
L1-7 (B)
V8 (J)
C5 (I)
MAV3 (J)
HEX1 (I)
HEX4 (I)
HEX3 (I)
HEX2 (I)
Preg2 (I)
Preg5 (I)
Preg3 (I)
Preg4 (I)
Preg1 (I)

Fig. S8

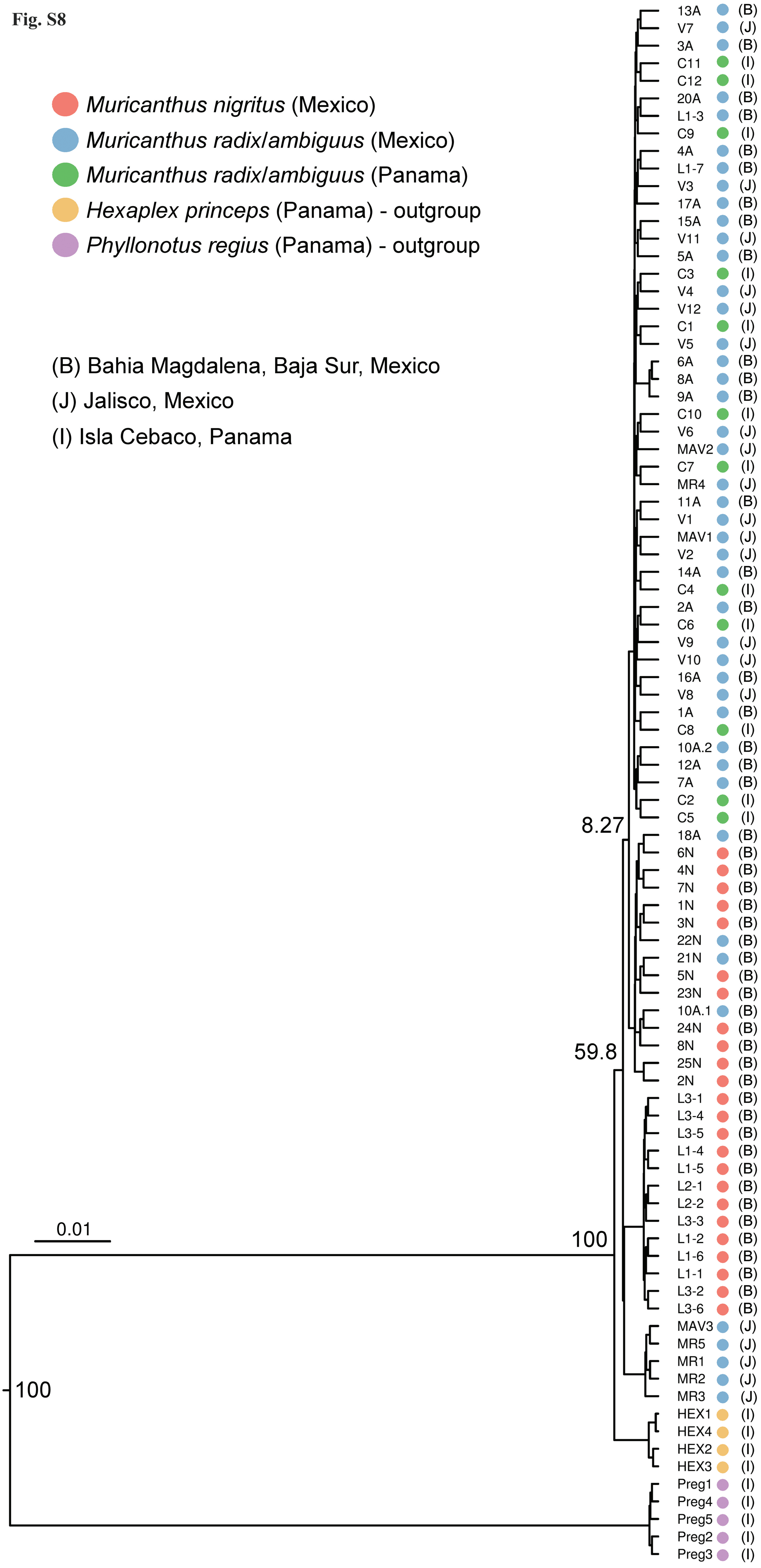

Muricanthus nigritus (Mexico)
Muricanthus radix/ambiguus (Mexico)
Muricanthus radix/ambiguus (Panama)
Hexaplex princeps (Panama) - outgroup
Phyllonotus regius (Panama) - outgroup
(B) Bahia Magdalena, Baja Sur, Mexico
(J) Jalisco, Mexico
(I) Isla Cebaco, Panama
0.01
8.27
59.8
100
100
13A (B)
V7 (J)
3A (B)
C11 (I)
C12 (I)
20A (B)
L1-3 (B)
C9 (I)
4A (B)
L1-7 (B)
V3 (J)
17A (B)
15A (B)
V11 (J)
5A (B)
C3 (I)
V4 (J)
V12 (J)
C1 (I)
V5 (J)
6A (B)
8A (B)
9A (B)
C10 (I)
V6 (J)
MAV2 (J)
C7 (I)
MR4 (J)
11A (B)
V1 (J)
MAV1 (J)
V2 (J)
14A (B)
C4 (I)
2A (B)
C6 (I)
V9 (J)
V10 (J)
16A (B)
V8 (J)
1A (B)
C8 (I)
10A.2 (B)
12A (B)
7A (B)
C2 (I)
C5 (I)
18A (B)
6N (B)
4N (B)
7N (B)
1N (B)
3N (B)
22N (B)
21N (B)
5N (B)
23N (B)
10A.1 (B)
24N (B)
8N (B)
25N (B)
2N (B)
L3-1 (B)
L3-4 (B)
L3-5 (B)
L1-4 (B)
L1-5 (B)
L2-1 (B)
L2-2 (B)
L3-3 (B)
L1-2 (B)
L1-6 (B)
L1-1 (B)
L3-2 (B)
L3-6 (B)
MAV3 (J)
MR5 (J)
MR1 (J)
MR2 (J)
MR3 (J)
HEX1 (I)
HEX4 (I)
HEX2 (I)
HEX3 (I)
Preg1 (I)
Preg4 (I)
Preg5 (I)
Preg2 (I)
Preg3 (I)

(a) **Fig. S9**

Nb of subsets	asap-score		P-val (rank)	W (rank)	Treshold dist.	Text
* 4	2.00		3.94e-03 (1)	1.47e-04 (3)	0.038773	list csv
2	3.00		5.53e-01 (4)	2.87e-04 (2)	0.105803	list csv
* 5	3.50		7.62e-01 (6)	4.39e-03 (1)	0.016012	list csv
* 6	4.00		4.11e-01 (3)	4.81e-05 (5)	0.010651	list csv
* 8	6.00		6.37e-01 (5)	2.24e-05 (7)	0.008350	list csv
* 7	6.50		8.32e-01 (7)	2.24e-05 (6)	0.009121	list csv
4	7.00		8.68e-01 (10)	5.86e-05 (4)	0.067564	list csv
57	7.50		4.30e-03 (2)	9.64e-06 (13)	0.000756	list csv
21	9.00		8.50e-01 (9)	1.36e-05 (9)	0.003028	list csv
34	10.50		9.46e-01 (13)	1.53e-05 (8)	0.001513	list csv

Number of subsets in this table equals to the number of primary species hypotheses in the respective partition

Histogram of distances [save]

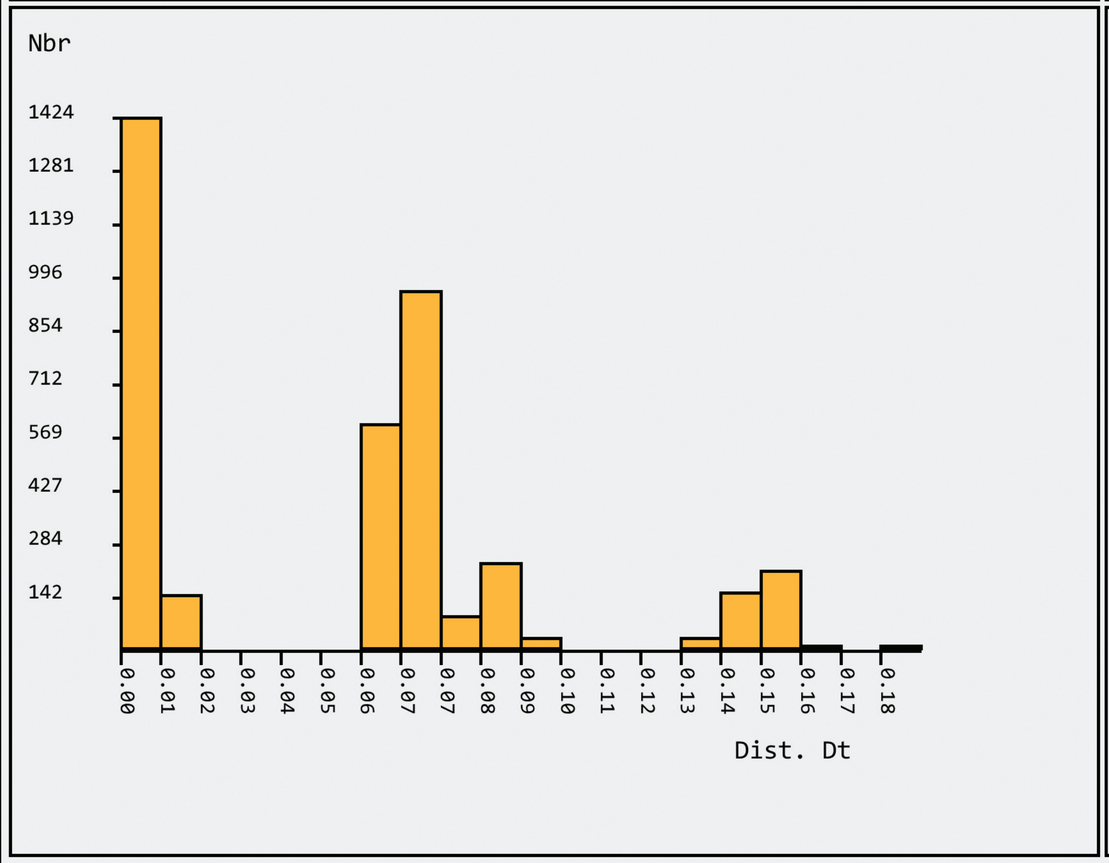

Ranked distances [save]

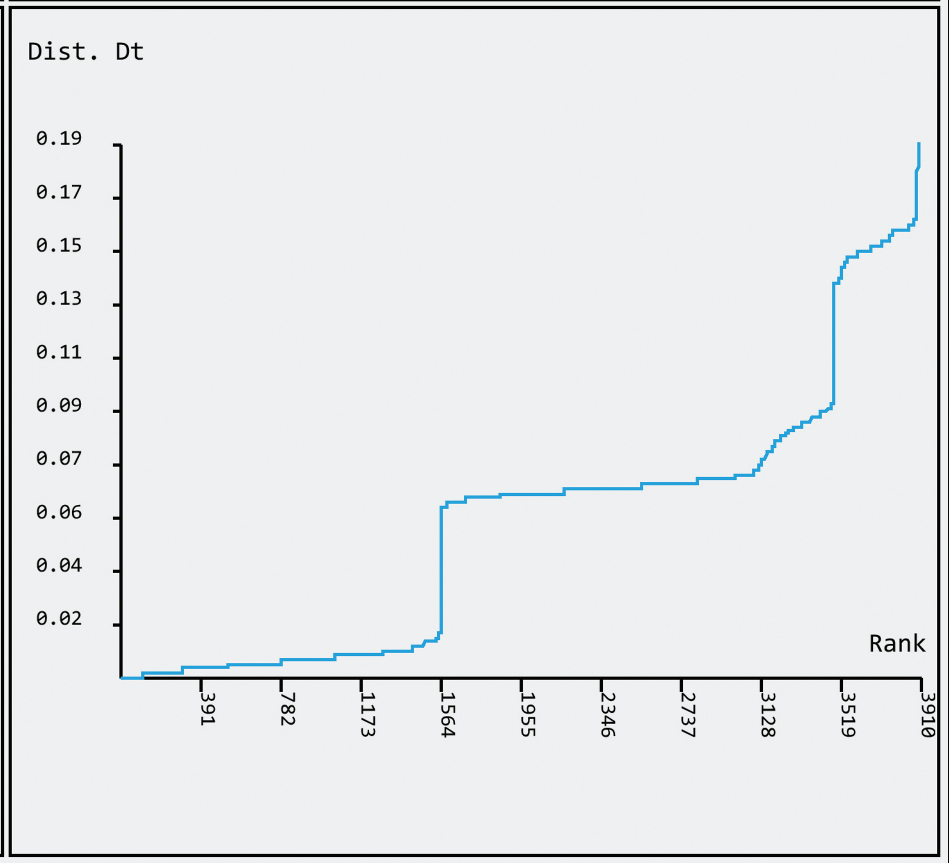

(b)

Nb of subsets	asap-score		P-val (rank)	W (rank)	Treshold dist.	Text
* 2	1.00		3.52e-03 (1)	1.56e-04 (1)	0.033412	list csv
* 3	2.50		8.26e-01 (3)	2.26e-05 (2)	0.008355	list csv
16	4.50		8.60e-01 (5)	1.37e-05 (4)	0.003028	list csv
49	5.00		4.18e-03 (2)	9.76e-06 (8)	0.000756	list csv
27	5.50		9.38e-01 (8)	1.56e-05 (3)	0.001513	list csv
23	7.00		9.08e-01 (7)	1.09e-05 (7)	0.002270	list csv
* 7	7.50		8.50e-01 (4)	8.75e-06 (11)	0.005307	list csv
* 5	8.00		9.02e-01 (6)	9.03e-06 (10)	0.006823	list csv
80	8.00		1.00e+00 (11)	1.30e-05 (5)	0.000000	list csv
8	8.00		1.00e+00 (10)	1.15e-05 (6)	0.004547	list csv

Number of subsets in this table equals to the number of primary species hypotheses in the respective partition

Histogram of distances [save]

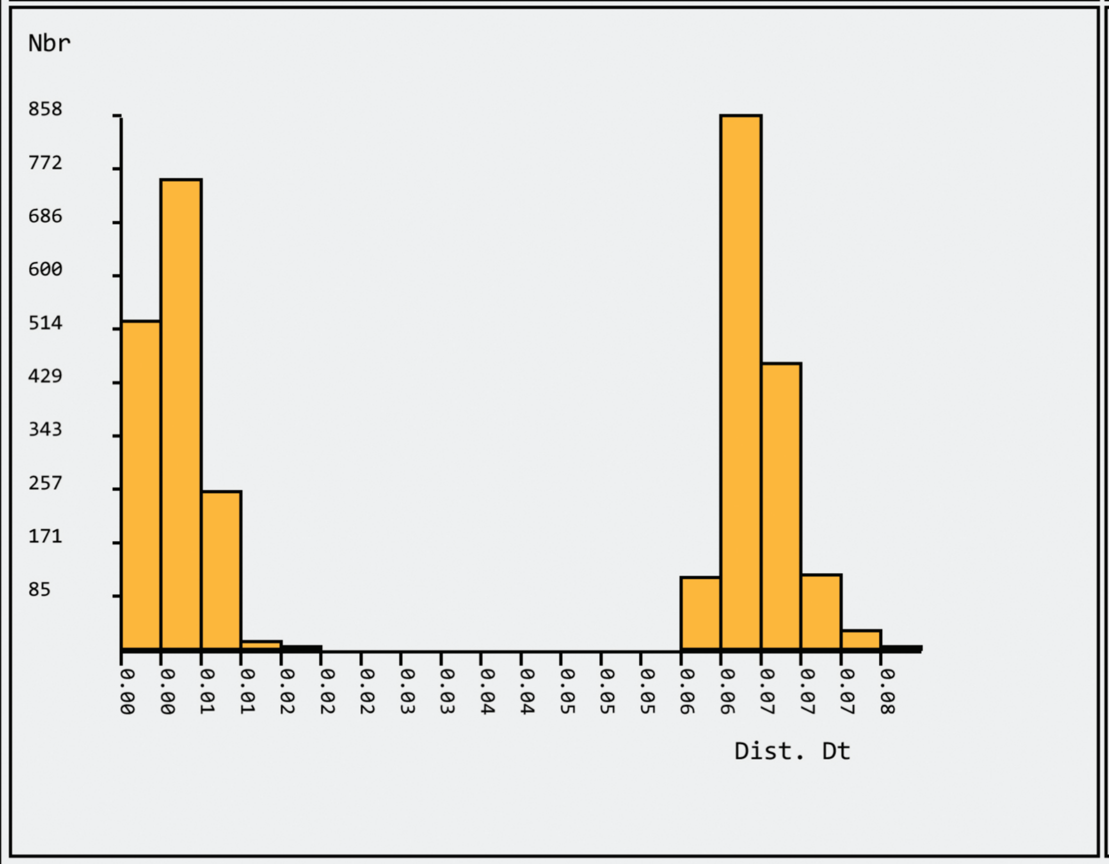

Ranked distances [save]

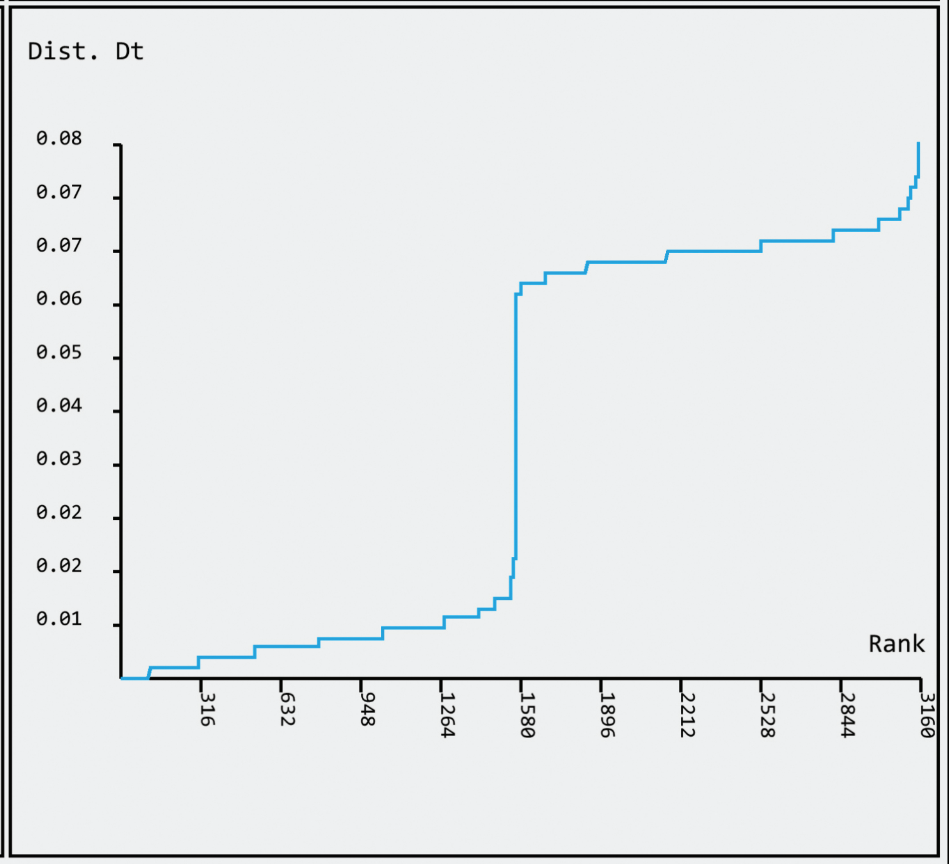

Supplement: Supplementary material 2 — Additional images [file zookeys-1239-281_article-143837__-s002.pdf]
